# Supplementary figures and images for: VolPy: Automated and scalable analysis pipelines for voltage imaging datasets
Source: PLoS Comput Biol. 2021 Apr 14;17(4):e1008806. doi: 10.1371/journal.pcbi.1008806 (PMC8075204; doi:10.1371/journal.pcbi.1008806)

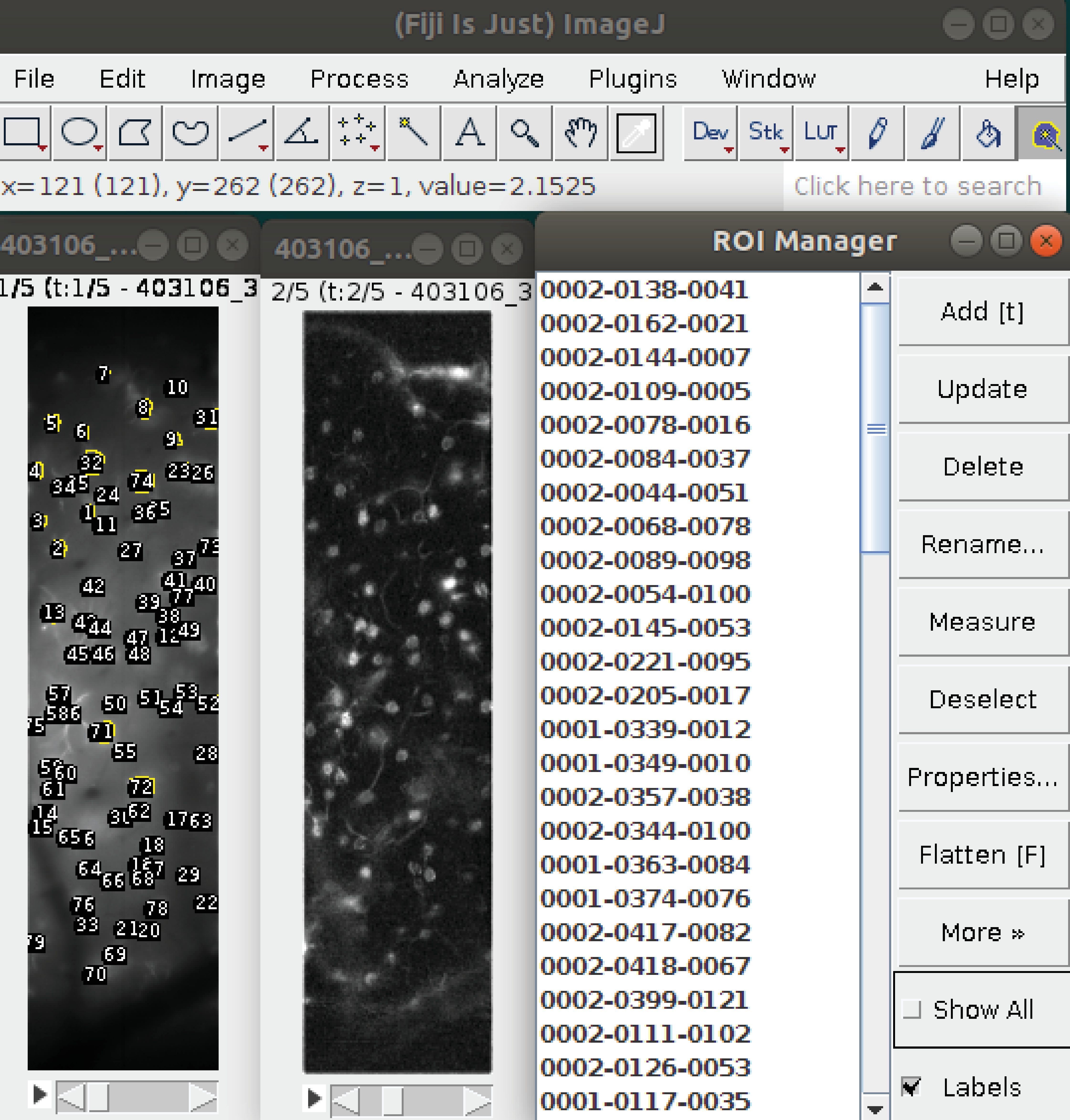

Supplement: S1 Fig — We selected neurons based on mean image (left), correlation image (mid). Three annotators marked the contours of neurons independently using ImageJ Cell Magic Wand tool plugin and showed selections in ROI manager (right). (TIF) [file pcbi.1008806.s002.tif]

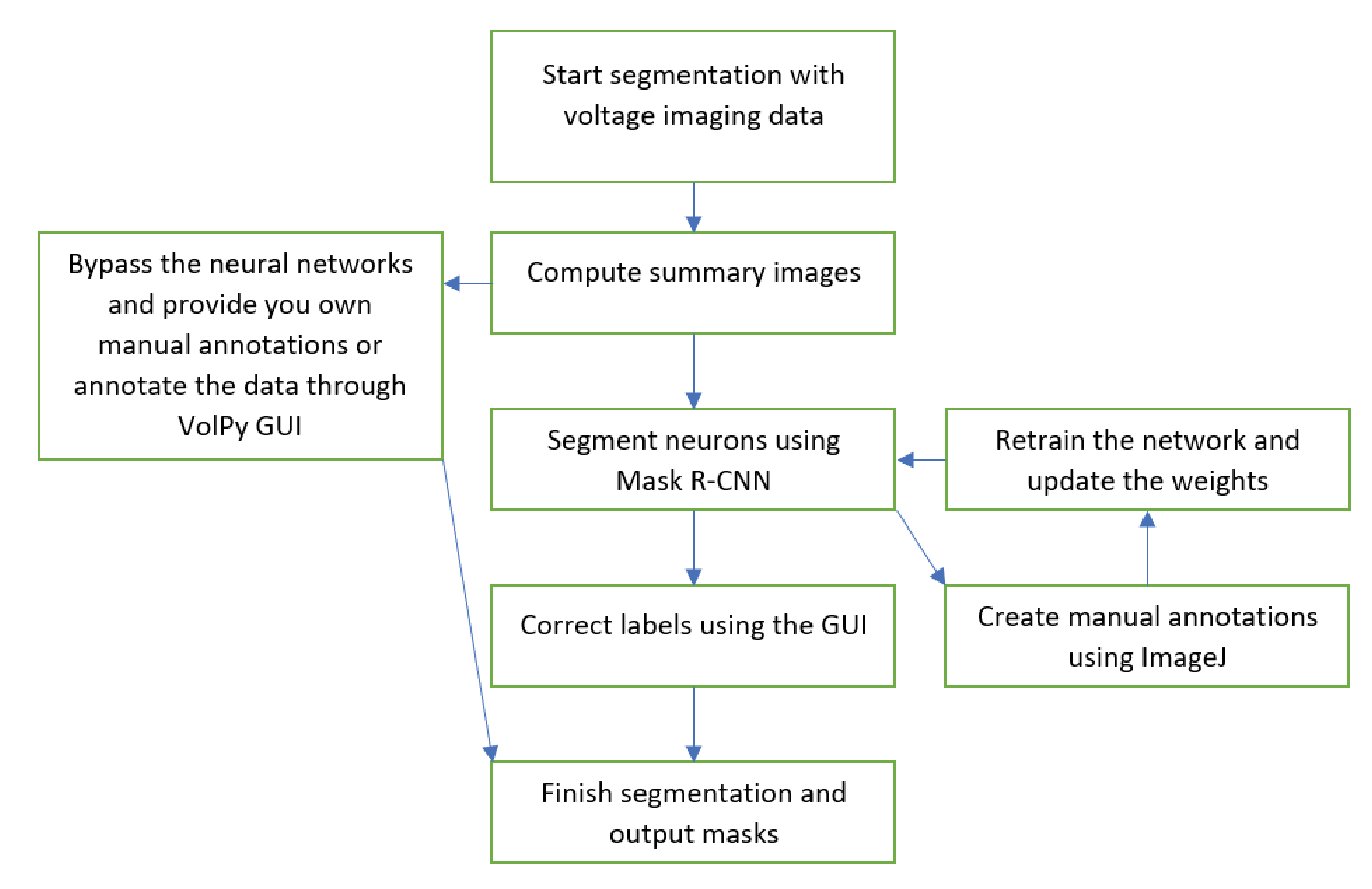

Supplement: S3 Fig — Summary images are computed from input voltage imaging movies. Subsequently masks of neurons can be provided in two ways. 1. Neurons can be segmented via a Mask R-CNN neural network trained on the three types of datasets presented in this paper (L1, TEG and HPC). The output labels can be further corrected by the VolPy GUI (See S1 Vid). If users are not satisfied with results of Mask R-CNN, they can manually annotate voltage imaging datasets using ImageJ. Such new annotations can then be used to retrain Mask R-CNN. Details for retraining Mask R-CNN are explained at the page https://github.com/flatironinstitute/CaImAn/wiki/Training-Mask-R-CNN 2. Users can also bypasss the Mask R-CNN step and choose to provide their own manual masks labelled either through other softwares or VolPy GUI. (TIF) [file pcbi.1008806.s004.tif]

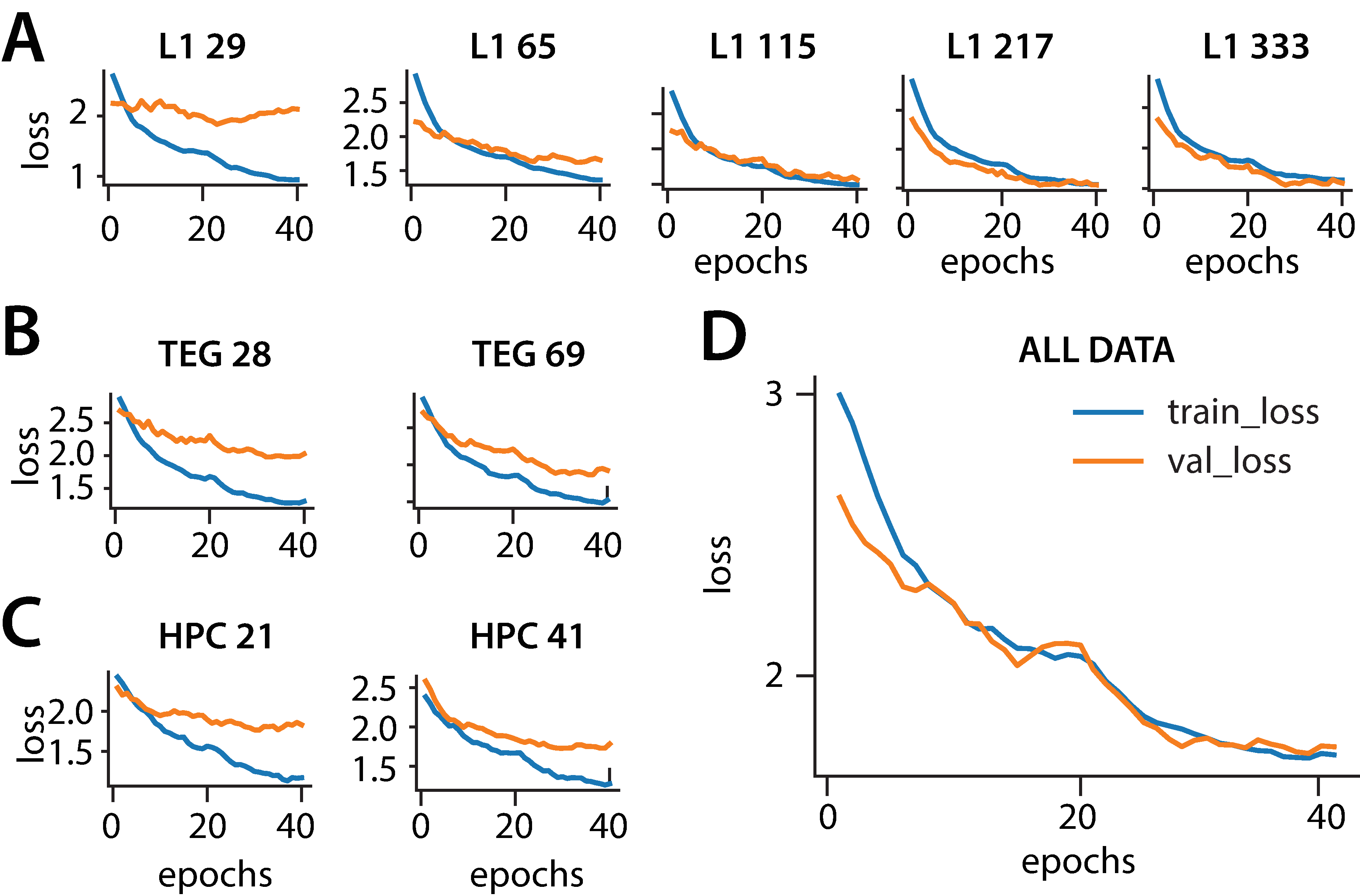

Supplement: S4 Fig — Learning curves corresponding to data in Fig 3D. Training (blue) and validation (orange) loss in function of training set size for L1 (A), TEG (B) and HPC (C) datasets. (D) For comparison, learning curves for training and validation set when training on all the datasets. (TIF) [file pcbi.1008806.s005.tif]

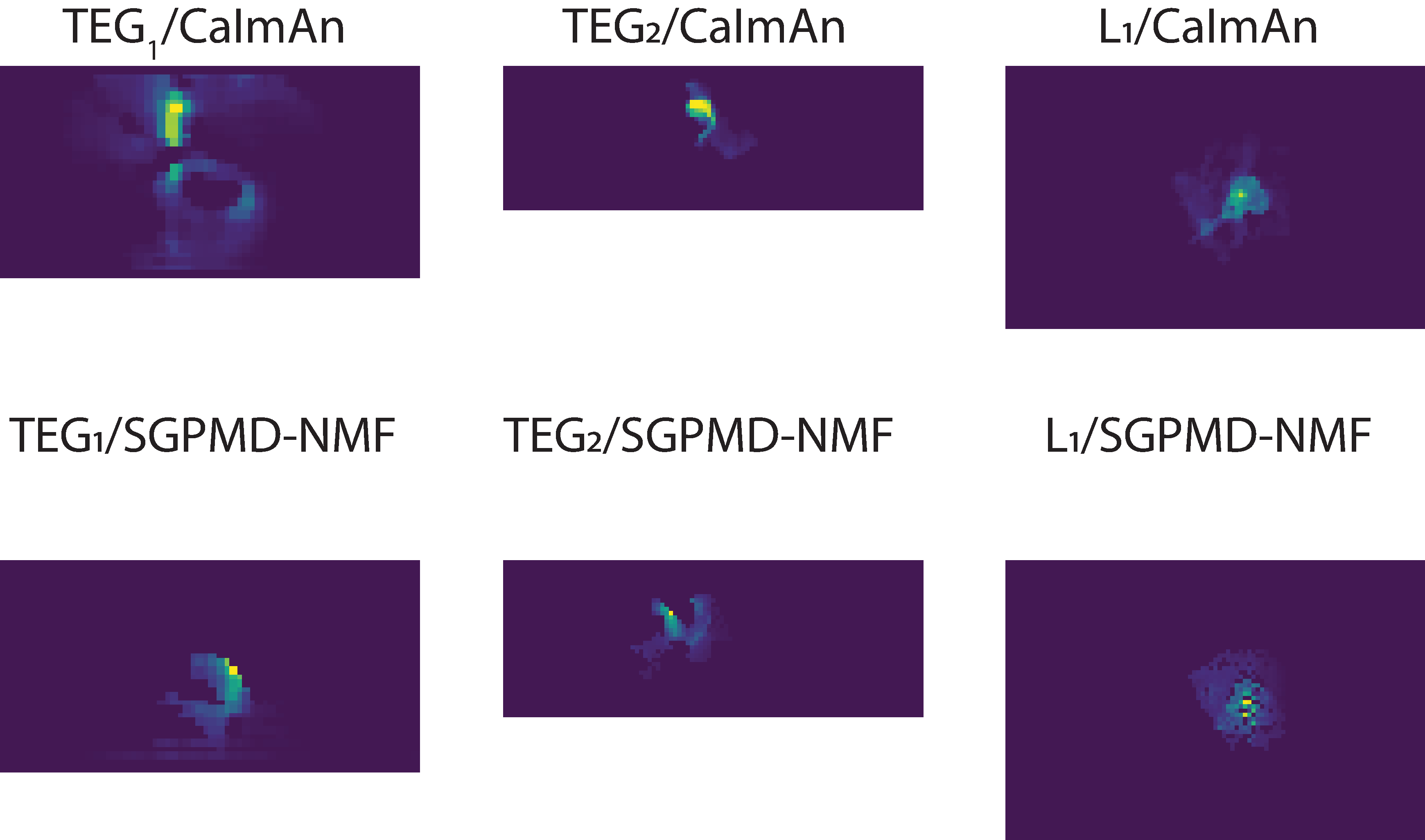

Supplement: S5 Fig — Spatial footprints extracted by CaImAn and SGPMD-NMF on the data reported in Fig 5A. (TIF) [file pcbi.1008806.s006.tif]
