# Supplementary material for: VolPy: Automated and scalable analysis pipelines for voltage imaging datasets
Source: PLoS Comput Biol. 2021 Apr 14;17(4):e1008806. doi: 10.1371/journal.pcbi.1008806 (PMC8075204; doi:10.1371/journal.pcbi.1008806)
Supplement: Attachment — Submitted filename: ResponseToReviewers.pdf [file pcbi.1008806.s011.pdf]

Dear Editor,

Thank you for taking the time to oversee our submission revision process. Below find our replies to the raised concerns and how we addressed them. Further, we would like to thank the reviewers and the editor for their thorough and thoughtful reviews. VolPy is a big project and the reviews touched upon almost all of its aspects leading to a large revision of our paper. We believe that our current submission addresses all the issues raised by reviewers. In summary the revised version of our paper includes (among many other improvements):

- A less biased corpus of annotations obtained by involving three independent labelers
- A thorough and systematic comparison with existing methods in detecting spikes using both simulated and real data.
- A more systematic description of the inner workings of VolPy, with more detailed descriptions of motion correction and spike extraction
- A systematic study on the scalability in terms of computational time and memory consumption of VolPy against comparable algorithms.
- A more detailed description of the image segmentation pipeline along with its failure modalities. We also included a graphical user interface to refine the results obtained via automatic segmentation, and a detailed protocol to annotate new datasets and retrain the network with custom data.

Concurrently with this submission we also released a new version of VolPy, that presents a simplified way to run the pipeline and refine the results at the different stages of analysis.

Please find our detailed responses below. Since our paper is long, we copied excerpts from the paper (text, figures etc) that directly address the reviewers' concerns. Please also note that since the paper changed substantially, the marked-up version of the paper obtained with latexdiff is very difficult to parse. We uploaded it anyway, since it was required. We tried to highlight in the response to reviewers the most relevant changes in the paper.

Both reviewers agree on the need for analysis tools for voltage-imaging data. Due to the nature of the underlying signals and the reporters, these data do pose a significant challenge for analysis, to detect real signals, disambiguate the cellular sources etc. This is obviously an evolving field since breakthroughs in reporters will change the requirements for analysis, hopefully making life easier. Both reviewers agree that the approaches taken in this article are reasonable given the data sets that they consider. The proposed pipeline is a collection of previously described approaches and techniques, not new techniques.

Both reviewers agree that the paper would have far higher impact if it included comparisons to existing methods, some of which have been tested on voltage imaging data. I agree and comparisons should be made to the tools mentioned by reviewer 1. Claims of validity of methods were based on a small number of annotators, so again, it is

crucial to check claims and expected behavior against other methods and revise either the methods or claims if and when unexpected results appear.

Both concerns are reasonable and we agree, they would make for a more impactful paper. We have addressed all of the raised concerns, as outlined below in response to reviewers.

There are also a large number of specific questions and concerns from reviewer 1, which must be addressed point by point.

We addressed all the questions and concerns by reviewers one and two.

Please also answer the following questions from the editor: 1. Were surrogate data sets used or can they be used to test validity of the methods? 2. Were model data sets with known ground truth spike times, cell identities, etc. constructed or used or can they be used to test the validity of methods?

We addressed questions 1 and 2 from the editor in:

- Reviewer 1. Main comments, Point 1, and Specific comments, point 6
- Reviewer 2 Main point 2.

We do not add here a duplicate of responses to compress an already long rebuttal letter.

Reviewer's Responses to Questions

Comments to the Authors:

Please note here if the review is uploaded as an attachment.

Reviewer #1:

The paper presents an automated analysis framework for Voltage imaging data alongside a collection of 24 manually annotated datasets. The framework performs motion correction, segmentation and spike extraction and has been incorporated in the CaImAn python codebase. Performance on the included datasets, especially L1 is impressive. This software pipeline along with the curated datasets will advance analysis of voltage imaging in the community.

We thank the reviewer for the thorough review, and the useful suggestions, which we hope to have satisfactorily addressed.

Main comments:

1. Why is there no comparison to other methods? While VolPy provides a complete pipeline, its intermediate results such as cell segmentation or spike extraction can be compared to other approaches. The authors make the point that NMF-based approaches are not suitable for Voltage imaging, however other CI approaches such as PCA/ICA,

Suite2p, ABLE, etc have less strict assumptions on the modeling of calcium imaging data than the NMF-based approaches the authors have published in the past. Have these methods been evaluated for voltage imaging?

The method in [13] has also been tested on voltage imaging – why is there no comparison? If results are indeed poor, then the authors can at least report that they attempted these approaches with poor performance. Comparison to demonstrate the authors have indeed improved the SpikePursuit algorithm should also be included.

Following the suggestions of the reviewer we compared VolPy thoroughly to other approaches, including calcium imaging analysis algorithms and [13]. In most cases some very specific preprocessing of the data was necessary to get the algorithms working. Also, no evaluated method, except SpikePursuit, had a means to automatically extract spikes. We therefore also implemented some basic spike extraction methods. In the text we included the following explanation regarding the methods we compared to:

Implementation of benchmarked algorithms

We compared *VolPy* against a set of other algorithms. Some of them could not directly be applied to voltage imaging, and therefore we had to introduce some modifications to adapt them. In what follows we describe how we deployed each of them.

CaImAn

CaImAn is a software package for the analysis of calcium imaging data [16] and can be found at the github repository `https://github.com/flatironinstitute/CaImAn`. For voltage imaging movie using indicator with reversed polarity (i.e. brighter for lower voltages, such as Voltron), vanilla *CaImAn* failed to retrieve reasonable spatial or temporal components because the NMF framework was unable to extract negative spikes of voltage signals. Just flipping the signal and removing the minimum of the whole movie also leads to poor performance. The best results were obtained by flipping the signal and removing the minimum value of each pixel. This helps *CaImAn* focus on the variance related to the voltage signal and not on baseline fluctuations. This modified movie can be processed via the standard *CaImAn* pipeline. We used the *greedy roi* method for spatial footprint initialization. We turned off the deconvolution step used for calcium signals, and instead we high-passed the temporal components with a 15Hz filter and applied a manual threshold to extract spikes.

For simulations, as movies were simulated with negative spikes, we processed them in the same way as we did for movies using Voltron indicator.

For voltage imaging movies using the paQuasar indicator, as they had positive spikes, we passed the original movie directly into the *CaImAn* pipeline without preprocessing and performed the same spike extraction step as mentioned before.

MeanROI

Region of interests were provided from ground truth masks beforehand. The MeanROI method extracts the voltage signal for each neuron by averaging the pixels within the provided masks. Depending on the signal polarity the trace can be flipped. Analogously to *CaImAn*, spikes are extracted by high-passing the signals with a 15Hz filter and manual thresholding.

Suite2P

Suite2p is a software package for the analysis of calcium imaging data [17]. We tested Suite2P on simulated datasets using the software available on github `https://github.com/MouseLand/suite2p`. To obtain good spatial footprints, we set the *overlapping* parameter to be True across simulations. When the spike amplitude was lower or equal to 0.01, we turned off the *sparse_mode* and when the spike amplitude was 0.005, we turned off the *connected* parameter. We flipped and high-passed the signals with a 15Hz filter and applied a manual threshold to extract spikes.

SGPMD-NMF

SGPMD-NMF is a set of software packages that can be deployed for the analysis of voltage imaging datasets [11, 12]. We obtained SGPMD-NMF from the Github repositories `https://github.com/adamcohenlab/invivo-imaging` and `https://github.com/m-xie/trefide.git`. We fed the original movie into the denoising step [11]. We flipped the output signal if it had reverse polarity and passed it to the demixing step, which outputs spatial footprints and temporal traces, along with subthreshold activities [12]. We high-passed the signals with a 15Hz filter and applied a manual threshold to extract spikes.

PCA-ICA

We implemented the PCA-ICA algorithm based on previous work on calcium imaging [14]. We chose the top 50 principal components in PCA and 15 components in spatial-temporal ICA. The parameter which controls the relative contribution of spatial and temporal information was set to 0.05. The output spatial components were updated after Gaussian smoothing and thresholding. Temporal signals were extracted as the weighted average of the updated spatial components. We flipped and high-passed the signals with a 15Hz filter and applied a manual threshold to extract spikes.

SpikePursuit

We recovered the original SpikePursuit implementation in Matlab from the github repository `https://github.com/KasparP/SpikePursuitMatlab` and ran it on simulated data and scalability tests. Ground truth masks were provided as the input and the algorithm used the *adaptive threshold* method to automatically select the optimal threshold and spikes times. For the scalability tests, parameters were set similarly to *VolPy*.

Spike Extraction

In this paper we tested the algorithms above and compared their performances against *VolPy*. Since only *VolPy* and SpikePursuit were able to extract spikes automatically, we modified the other algorithms to automate the spike detection process, and thus provide a direct comparison with *VolPy*. The general process to extract spikes was to high-pass the temporal components extracted by each algorithm with a 15Hz filter, and then to apply a manual threshold to extract spikes. The manual threshold was an estimated level of standard deviation of the signal based on the negative portion of the signal (similarly to SNR measure for calcium traces in [16]). In simulations, instead of picking a single manual threshold (for example 3.0), for each spike amplitude value we performed a grid search (range 2.0-4.0 with interval 0.1), and selected the threshold outputting the best average F_1 score across all neurons. In simultaneous electrophysiology and voltage imaging datasets and in-vivo datasets, the thresholds for *CaImAn* , MeanROI and SGPMD-NMF were chosen manually. In in-vivo datasets, the SpNR was computed only on the intersection of detected spikes among different algorithms.

We compared these algorithms on simulated datasets, voltage imaging with simultaneous electrophysiology datasets and in-vivo datasets using two metrics: the precision/recall framework and Spike To Noise Ratio (SpNR).

Voltage imaging datasets

In-vivo datasets

The datasets we employed were all previously published: Recordings from the tegmental area of larval zebrafish (TEG) and mouse L1 cortex (L1) are described in [5]; Recordings from mouse hippocampus (HPC) are described in [6]. For details about animal protocols and data acquisition refer to the original papers. The name, size and number of labeled neurons for each dataset is reported in Table 2.

Voltage imaging with simultaneous electrophysiology datasets

The simultaneous imaging and electrophysiological data presented in this paper were previously published in [5]. Two of them were extracellular recordings from the TEG area of larval zebrafish, and one intracellular recording from mouse L1 cortex. For details about animal protocol and acquisition refer to the original paper.

Simulated datasets

We generated simulated voltage imaging movies modelled upon the L1 dataset examples (Figure 4a). Fluorescence traces were obtained by combining the following components: (i) Spikes times were simulated with an inter spike interval uniformly distributed between 0.1 and 0.2 seconds; (ii) Fluorescence signals associated to spikes were obtained by convolving with a kernel matching the dynamics of Voltron signal in L1 neurons; (iii) Subthreshold activity was simulated by applying a Gaussian filter to white noise; (iv) Fluorescence signals of spikes and subthreshold activity were flipped to match the reverse polarity of the Voltron indicator; (v) To simulate photo-bleaching, the resulting fluorescence signal was modulated with an exponential decaying with a 2500s time constant.

Spatial footprints were simulated as ring shaped and real-valued masks with a small process protruding at different angles (Figure 4a). The shape and size was matched to L1 neurons in real data. The signal associated to each neuron within the movie was obtained by multiplying the simulated fluorescence signal times the spatial footprint. The sum of all neurons represented the imaging movie without background. In order to generate a realistic background signal, we summed the movie without background with a 50x50 pixels patch with no visible neurons from a motion corrected L1 dataset movie. When summing neurons and background, the baseline fluorescence of neurons (brightness) was selected to approximately correspond to *in-vivo* recordings. The spike amplitude was adjusted by changing the amplitude of fluorescence signals with the baseline fluorescence fixed (i.e. changing DF with F fixed). We added out of focus signals for different neurons on our simulated data. The out of focus signal was generated by multiplying the signal of each neuron times a spatial weight computed by applying a Gaussian filter on a randomly selected pixel in the FOV. Finally, white noise was added to every voxel in the simulated data.

For experiments with non-overlapping neurons, we tested neurons with spike

amplitudes 0.05, 0.075, 0.1, 0.125, 0.15, 0.175. 0.2. For overlapping cases, we tested neurons with overlapping areas 0%, 6%, 19%, 26% and 35%, and spike amplitudes 0.075, 0.125 and 0.175.

Spike-to-noise-ratio

In order to compare the performance of different algorithms when no ground truth data is available, we have defined a metric to quantify how well an algorithm was able to increase the detectability of spikes in voltage imaging traces. When comparing two algorithms, the *Spike-to-noise-ratio* (SpNR) is computed by identifying the set of spikes which were detected by both algorithms, and then calculate, for each denoised trace independently, the ratio between the average spike amplitude and the noise estimated as the standard deviation of the negative portions of the 15Hz high-pass filtered signal. We believe this metric is independent on thresholding methods and should provide an unbiased estimate of an algorithm denoising capability.

For the simulations, we compared VolPy to all benchmarked algorithms mentioned above. The results showed that in most cases, especially in the low SNR settings, Volpy performed better than other methods in F1 score and SpNR. Since the spike extraction accuracy (F1 score) changes with different thresholds, we selected for each algorithm (including VolPy) a threshold which outputs the best F1 score given spike amplitude. This threshold provides the best result an algorithm could ideally get (Fig 4c left). SpikePursuit adopts an adaptive thresholding method which does not need manual thresholding and was therefore directly compared to VolPy with automatic threshold (Fig 4c right). The performance of VolPy was slightly better than SpikePursuit mainly because we introduced a more robust way to remove the background. We also compared VolPy with CaImAn, MeanROI and SGPMD-NMF on voltage data with simultaneous electrophysiology. Only VolPy and MeanROI were able to extract reasonable fluorescence traces on two fish datasets. We believe that CaImAn and SGPMD-NMF failed because neurons in these datasets were not firing with homogeneous spatial footprints, as one can observe from S4 Vid. Also, the denoising step in SGPMD-NMF sometimes reduced the SNR on these noisy datasets. For other voltage imaging datasets (L1, TEG, HPC), VolPy showed a better SpNR compared to CaImAn, MeanROI and SGPMD-NMF. These comparisons were reported in Figs 4,5.

Since some of the algorithms we compared against *VolPy* require a substantial amount of work and tweaking to run, often entailing to execute multiple portions of code in different languages, we did not carry out multiple runs of the simulations. We do think the results across simulations and real datasets display sufficient evidence of the superiority of VolPy.

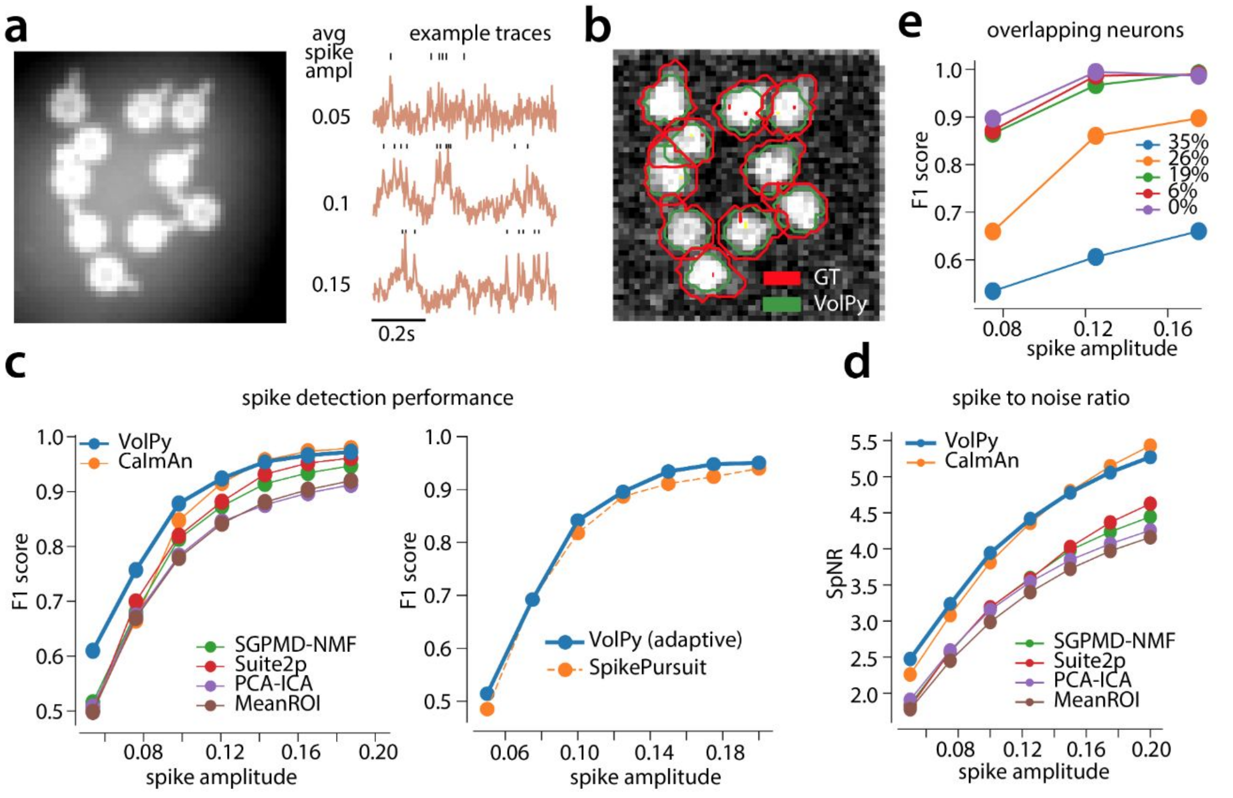

Fig 4. Evaluation of *VolPy* on simulated data. (a) Example of simulated data. Left. Average of movie across time. Right. Three example traces with different average spike amplitude. Higher spike amplitude are associated with higher signal to noise ratio. (b) The result of Mask R-CNN in segmenting the simulated movie (0.1 spike amplitude) laying over the correlation image. (c-d) Performance of *VolPy*, *CaImAn*, SGPMD-NMF, Suite2P, SpikePursuit, PCA-ICA and MeanROI on simulated data. (c) Average F_1 score against ground truth in function of spike amplitude. (Left) All algorithms (including *VolPy*) were evaluated with the optimal threshold. (Right) Comparison with SpikePursuit, *adaptive threshold* in both cases. (d) Spike-to-noise ratio (SpNR) in function of spike amplitude. (e) Evaluation of *VolPy* on overlapping neurons. Average F_1 score detecting spikes in function of spike amplitude and overlap between two neurons.

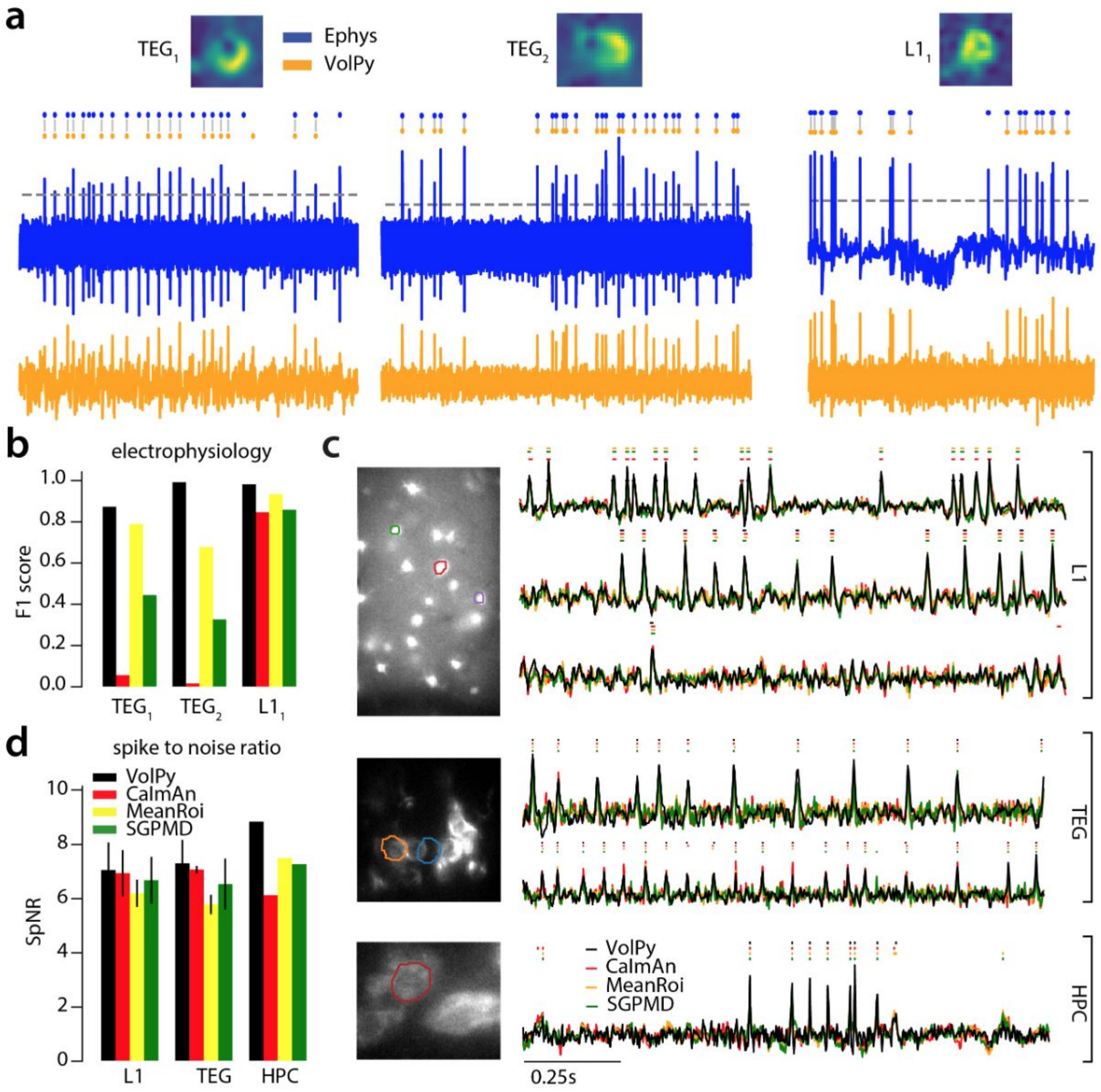

Fig 5. ***VolPy* performance on real data.** (a-b) Evaluation of *VolPy* spike extraction performance against simultaneous electrophysiology. (a) Three neurons, two from larval zebrafish TEG area (TEG_1 and TEG_2) and one from mouse L1 ($L1_1$), for which we had available both electrophysiology and imaging. Top. Spatial footprint extracted by *VolPy*. Middle. Ground truth spikes from electrophysiology (blue) and spikes extracted by *VolPy* (orange), gray vertical lines indicate matched spikes. Bottom. Electrophysiology (blue, top) and fluorescence signal denoised by *VolPy* (bottom, orange). (b) We compared the performance of *VolPy*, *CaImAn*, MeanROI, and SGPMD-NMF in retrieving spikes on the three neurons in (a). (c) Examples of trace extraction results for *VolPy*, *CaImAn*, MeanROI, and SGPMD-NMF. On the left mean image overlaid to example neurons (top L1, middle TEG, bottom HPC). On the right traces and inferred spikes for datasets L1 (top three traces), TEG (traces 4-5 from top) and HPC (bottom trace). (d) Spike to noise ratio (SpNR) for each considered algorithm and dataset type.

Although there is an only mild increment in accuracy of detecting spikes versus SpikePursuit, the computational performance of VolPy is significantly improved over SpikePursuit (Fig 6 c, d). Volpy was 3.5X faster than SpikePursuit and consumed 3X less memory. Simulation results showed that VolPy with adaptive and simple threshold outperforms SpikePursuit. Besides, VolPy was packaged into a usable, documented and maintained open source package, already popular within the community. Finally, VolPy was more than 10X faster and used much less memory compared to SGPMD-NMF.

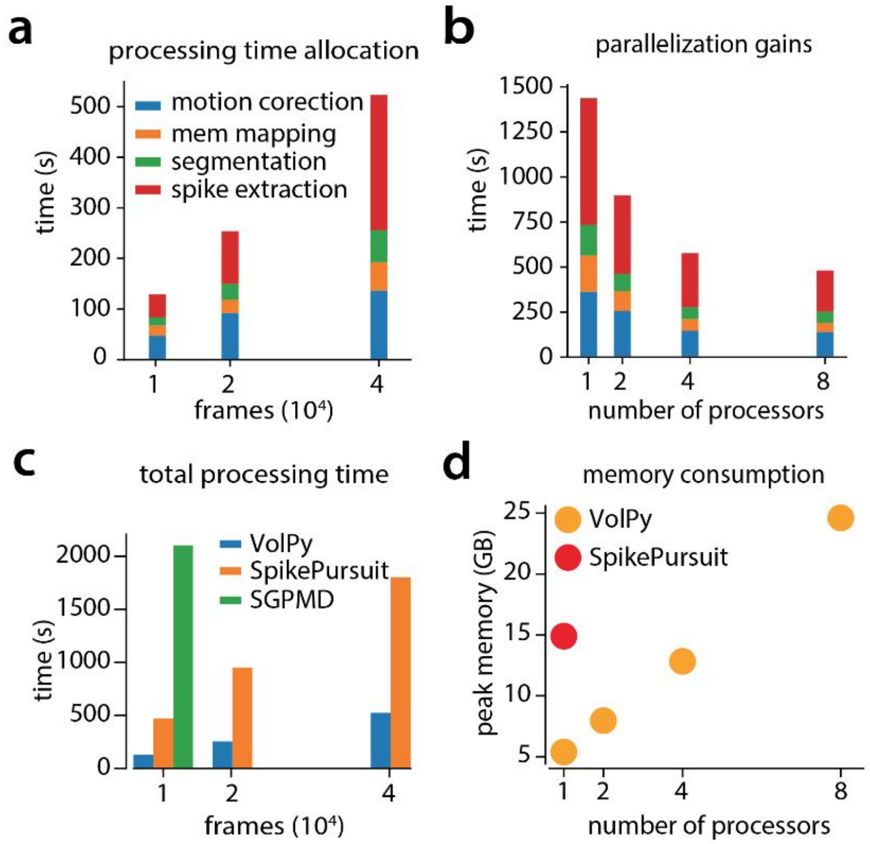

Fig 6. Evaluation of *VolPy* scalability. *VolPy* scalability was evaluated based on a 512x128 pixels movie with 75 annotated neurons. (a) Processing time allocation of *VolPy* with 10000, 20000 and 40000 frames using 8 processors. (b) Processing time of *VolPy* on 40000 frames with 1, 2, 4 and 8 processors. (c) Comparison of performance among *VolPy*(8 processors), SpikePursuit and SGPMD-NMF with 10000, 20000 and 40000 frames. (d) Peak memory usage of *VolPy* and SpikePursuit on 40000 frames. Since *VolPy* supports parallelization we reported memory usage with 1, 2, 4 and 8 processors.

2. Why were only 2 annotators used? What was the level of agreement between them?

We thank the reviewer for the opportunity to improve the paper. We re-annotated datasets with three independent annotators and different strategies for selecting neurons only based on mean

and correlation images. We assessed the degree of agreement between annotators. We explained these points in the text as follows:

Creation of a corpus of annotated datasets

To date there are no established annotated datasets for single cell localization and/or segmentation in cellular-resolution voltage imaging. Towards filling this gap, and with the goal of developing new supervised algorithms, we generated a corpus of 24 manually segmented datasets (Ground truth, GT) by combining annotations from three independent human labelers. To provide annotations, human labelers relied upon two

summary images (mean and local correlation images, Figure 1b,c Figure S1), which were built as follows:
Mean image: We averaged the movie across time for each pixel yielding a 2D image. We normalized the 2D image by subtracting the mean of its pixels and dividing by the standard deviation of its pixels. The normalization step enables different datasets to share the same scale as the input to the segmentation step.
Correlation image: The correlation image is a variation of that implemented in [20]. After removing the baseline of the movie by high-pass filtering, we averaged the temporal correlation of each pixel with its eight neighbor pixels yielding another 2D image. The resulting image was then mean-subtracted and divided by its standard deviation.

Guided by these visual cues, three annotators marked the contours of neurons using the FIJI ImageJ Cell Magic Wand tool plugin [21] (Figure S1). Labelers were trained using a test dataset and instructed to look for ring or circle-shaped structures which were clear on either the mean or the correlation image. An exception to this rule were blood vessels perpendicular to the imaging plane, which looked like dark circles in the mean image and bright circles in the correlation image. We then generated a consensus ground truth by combining the three annotations. For a neuron to be included in the consensus ground truth, it either had to be selected by two or more annotators, or all annotators had to agree on accepting it in a separate follow-up session. The finally selected pixels associated to a consensus mask was selected more based on masks provided by the most experienced annotators of the three. Summary information about the annotated datasets is reported in Table 1.

c

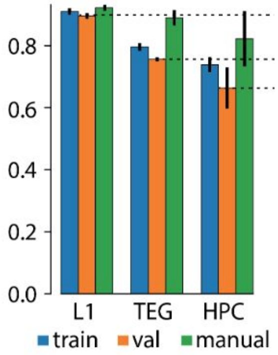

In Fig 3c, we compared the performance of the VolPy and the performance of the annotators. We regarded the combined annotations as ground truth and compared each human annotation against it. The level of agreement was measured as F1 score. The level of agreement also represented a measure of the difficulty of each dataset. For the L1 datasets, the average F1 score of the human annotation was 0.92±0.01; for the TEG datasets, 0.89±0.02; and for the HPC datasets,

0.82±0.09. Human annotators achieved high agreement on the L1 datasets, lesser on the TEG datasets and the least agreement on the HPC datasets.

3. Pages 7-8: multiple parameters and algorithmic choices are made in the processing and spike time estimation algorithms. Can the authors provide some insight or motivation to the selection parameters and heuristics used? For example why are 8 principal components used to estimate the background in line 180?

In our experience, many of the largest principal components describe structured global noise in voltage recordings. We chose to subtract the largest 8 components because subtracting fewer components would remove less spurious variants, while subtracting many more components would risk subtracting neuronal signals. We admit that this is a rudimentary denoising method, but it is simple to implement and effective. We have clarified this in the text.

$$\boldsymbol{\beta} = (U_b^T U_b + \lambda_b \|U_b\|_F^2 I)^{-1} U_b^T \mathbf{t}, \qquad (3)$$

where U_b is the first n_{pc} (default is 8) components of U, λ_b (default is 0.01) is the regularization strength and β is the estimated ridge regression coefficients. In our experience, many of the largest principal components describe structured global noise in

voltage recordings. We choose to subtract the largest 8 components because on real datasets subtracting fewer components would remove less spurious variants, while subtracting many more components would risk subtracting neuronal signals. Compared to SpikePursuit, we add an L_2 regularizer to penalize large regression coefficients caused by small components of background pixels with signals bleeding through from the neuron of interest. This provides more reliable results compared to the original linear regression method (see Results section). We choose λ_b to be 0.01 because we find that strong regularization strength (greater or equal to 0.1) will not help subtract the background from the signal while small regularization strength will not be able to penalize large regression coefficients enough. We next subtract the background signal from the trace $\mathbf{t}$ (③ in Fig 2b):

Do the authors have a model for the spike detection? The algorithm as described on top of page 8 is a collection of multiple processing steps without motivation.

- **Noise is estimated twice. Line 192 says peaks are 3.5 times the noise level, while line 203 states 4 times the noise level.**
- **Are the spikes detected using the matched filter or using the noise threshold?**
- **This subsection can be better organized to guide the reader so that the method is clear. For clarity perhaps the pseudo-code should be included here and not in the supplementary?**

We thank the reviewer for the suggestion and we think that indeed we were not precise in discussing the above points. We hope to have addressed in detail the above points in the following sections of the paper. In summary:

- We described the model underlying spike detection (lines 259-268)
- We specified in more details the two different portions of the algorithms when spikes are extracted and noise is estimated
- We clarified that the threshold is used to extract events after the matched filtering
- We added pseudocodes for the described function directly in the main text and further organized in section the text

Our spike extraction model is based on the idea of matched filters [22, 34]. A matched filter is an optimal linear filter for the detection of a given template from the signal under the hypothesis of additive white Gaussian noise. It has the goal of estimating the location of a given known waveform within a noisy signal while maximizing the SNR. Template matching is performed by correlating a template waveform with the noisy signal. As a consequence, we need two rounds of spike detection. The former is required to prewhiten the signal and form the template waveform. This is used to perform matched filtering by cross-correlating it and the prewhitened trace (matched-filtering). The latter consists in identifying and extracting the peaks from the whitened matched filter trace.

To threshold and extract spikes from the filtered signal $\mathbf{t_s}$ we provide two methods, *adaptive* and *simple threshold*. The *adaptive threshold* method selects a threshold (h) based on the distribution of local maxima, $P_{max}(x)$, approximated by kernel density estimation. The symmetrization of $P_{max}(x)$ around the median μ is used to approximate the noise distribution of peaks.

$$\widehat{P}_{noise}(\mu + x) = P_{max}(\mu - |x|) \tag{5}$$

The two distributions are then combined to estimate the threshold by minimizing the function:

$$h = \underset{h \in \mathbb{R}}{\arg\max} \Big(\Big(\int_h^\infty P_{max}(x)\, dx \Big)^p - \Big(\Big(\int_h^\infty \widehat{P_{noise}\, dx} \Big)^p \Big), \tag{6}$$

where p sets the stringency of the discrimination, reflecting a trade off between the benefit of including more (lower-amplitude) spikes in defining the template, and the cost of including additional noise spikes. Smaller values of p result in a more stringent threshold. Although the assumption that the noise distribution of peaks is symmetric around the median μ is not always fulfilled, experiments with ground truth and simulated data demonstrate that this approach is effective. We set $p = 0.25$ for the first round of spike detection. In our experiments, manual tuning of p was not needed for later stages of the algorithm to identify improved spatial and temporal filters.

In *VolPy*, we provide a second thresholding method, *simple threshold*, which solely relies on the noise level estimation. *simple threshold* only considers values below the median of the filtered trace to estimate the noise level σ. Only peaks larger than l (default as 3.5) times the noise level σ are selected. This method too assumes that the distribution of noise is symmetric around the median. In rigorous terms, the assumption of symmetric distribution is more realistic on the filtered signal than on peak heights.

Our rationale to introduce a second thresholding method is to help users with a more intuitive parameter to explore in the cases of *adaptive threshold* failure.

After the first round of spike detection, a spike template $\mathbf{z} \in \mathbb{R}^{2\tau+1}$ is computed by averaging the waveforms of the extracted peaks:

$$\mathbf{z}(t') = \frac{1}{n(\mathbf{s})} \sum_{t \in \mathbf{s}} \mathbf{t_s}(t + t') : t' \in [-\tau, \tau], t' \in \mathbb{Z} \tag{7}$$

where $\mathbf{s}$ is the set of spikes, $n(\mathbf{s})$ is the total number of spikes (⑤ in Fig 2b) and τ the waveform half size with default time bin of 20 ms (that is 8 frames if the movie was recorded at 400 Hz). Subsequently, a whitened matched filter [22] is used to enhance spikes with shape similar to the template (⑥ in Fig 2b). This operation is composed of two steps: (i) the signal is prewhitened in the frequency domain based on the noise spectrum estimated by the Welch method. The prewhitened signal has noise distribution similar to the white noise which is important as the matched filter is an optimal linear filter when the signal has additive white Gaussian noise. (ii) a new template $\mathbf{z}' \in \mathbb{R}^{2\tau+1}$ is computed from the prewhitened signal (equation 7) and template matching is performed by computing the cross-correlation between the prewhitened signal and the new template $\mathbf{z}'$. This final signal has peaks associated to spikes enhanced with respect to the original trace.

After the whitened matched filtering operation, a second round of spike detection using *adaptive/simple threshold* is carried out. While in the first round of spike detection p is set to 0.25 in order to avoid False Positives and gather spikes with high confidence to build a representative template, during the second round we aim to maximize F_1 score, and therefore set $p = 0.5$. When using the *simple threshold*, a threshold of 3.0 is used by default for a second round of spike detection. The newly detected spikes are transformed into a spike train $\mathbf{q} \in \mathbb{R}^T$. This new spike train is used to reconstruct a denoised version of the original signal, by convolving $\mathbf{q}$ with the template $\mathbf{z}$:

$$\mathbf{t_{rec}} = \mathbf{q} * \mathbf{z} \quad \text{where } \mathbf{q}(t) = \begin{cases} 1 & \text{if there is a spike at time t} \\ 0 & \text{otherwise} \end{cases} \tag{8}$$

Spatial filter refinement (⑦ in Fig 2b): The second step is to refine the spatial filter. The updated spatial filter is computed by regressing the reconstructed trace $\mathbf{t_{rec}}$ on the high-passed movie Y_h through Ridge regression:

$$\mathbf{w} = (Y_h^T Y_h + \lambda_w \|Y_h\|_F^2 I)^{-1} Y_h^T \mathbf{t_{rec}}, \tag{9}$$

where λ_w (default is 0.01) is the regularization strength. Instead of solving the ridge regression problem in its analytical form as SpikePursuit, we apply an iterative and efficient algorithm [35] implemented in the Scikit-Learn package ('lsqr') for better time performance. Subsequently, the weighted average of the movie with the refined spatial filter is used as the updated temporal trace for the following iteration:

$$\mathbf{t} = Y_h \mathbf{w} \tag{10}$$

For the final round, the spatial filter and the temporal trace are not updated.

Subthreshold activity extraction (⑧ in Fig 2b): After three iterations of spike time estimation (in our experience this was generally sufficient to converge to a stable solution) and spatial filter refinement, the subthreshold activity is extracted. First, a residual signal is computed by subtracting the reconstructed trace from the temporal trace $\mathbf{t_{res}} = \mathbf{t} - \mathbf{t_{rec}}$. Second, A 5th order Butterworth low-pass filter ($f_c = 20Hz$ by default) is applied on the residual trace $\mathbf{t_{res}}$.

Locality test: A locality test is performed finally to evaluate whether the reconstructed signal represents the original ROI or is contaminated by neighboring structures. First, we compute the correlation between the reconstructed signal $\mathbf{t_{rec}}$ and each pixel in the movie context region Y_h. Second, we check whether the pixels with maximal correlation are inside the original region of interest S or not. In case this did not happen, it would mean that the extracted signal represents other surrounding structures, and therefore it is discarded.

Importantly, inactive neurons are generally identified by the segmentation algorithm, but given the absence of spikes the spatial filters might not match structures internal to the provided masks, thereby failing the locality test. Therefore, inactive neurons with signals not representing the ROI can be removed since they fail locality tests.

Algorithm 1 **Trace Denoising and Spike Extraction**

Require: Movie in the context region $Y \in \mathbb{R}^{T \times N}$, where T is number of frames and N is number of pixels in the context region, the set of pixels in the region of interest S, the set of pixels in the local background B, the number of selected background principal components n_{pc}, the Ridge regression regularization coefficients λ_b, λ_w, the number of iterations K, and remaining parameters *params*

if $\textsc{ReversePolarityIndicator} = 1$ **then**
  $Y \leftarrow Y \cdot (-1)$
end if
$Y_h \leftarrow \textsc{HighPassFilter}(Y, \textit{params})$ ▷ Correct for photobleaching
if $\mathbf{w}$ is None **then**
  $\mathbf{t} \leftarrow \frac{1}{n(S)} \sum_{x \in S} Y_h(:, x)$ ▷ Averaging the signal across pixels in ROI
else
  $\mathbf{t} \leftarrow Y_h \mathbf{w}$ ▷ Compute weighted average across all pixels inside the context region
end if
$Y_b \leftarrow Y_h(:, B)$ ▷ Extract the background movie
$U, \Sigma, V \leftarrow \text{SVD}(Y_b)$ ▷ Compute the singular value decompostion of Y_b
$U_b = U(:, 1 : n_{pc})$
for $k \leftarrow 1 : K$ **do**
  $\boldsymbol{\beta} \leftarrow (U_b^T U_b + \lambda_b \|U_b\|_F^2 I)^{-1} U_b^T \mathbf{t}$ ▷ Ridge regression to remove background
  $\mathbf{t} \leftarrow \mathbf{t} - U_b \boldsymbol{\beta}$
  $\mathbf{t_s}, \mathbf{s}, \mathbf{t_{rec}}, \mathbf{z} \leftarrow \textsc{DenoiseSpikes}(\mathbf{t}, \textit{params})$ ▷ See Algorithm 2. Compute trace after whitened matched filter $\mathbf{t_s}$, spike time $\mathbf{s}$, reconstructed trace $\mathbf{t_{rec}}$ and spike template $\mathbf{z}$
  if $k < K$ **then**
    $\mathbf{w} \leftarrow (Y_h^T Y_h + \lambda_w \|Y_h\|_F^2 I)^{-1} Y_h^T \mathbf{t_{rec}}$ ▷ Refine spatial filter
    $\mathbf{t} \leftarrow Y_h \mathbf{w}$
  end if
end for
$\mathbf{t_{sub}} \leftarrow \textsc{LowPassFilter}((\mathbf{t} - \mathbf{t_{rec}}), \textit{params})$ ▷ Extract subthreshold activity
$m \leftarrow \textsc{ArgMax}(Y_h^T \mathbf{t_{rec}})$ ▷ Locality test
if $m \in S$ **then**
  $loc \leftarrow 1$
else
  $loc \leftarrow 0$
end if
return $\mathbf{t_s}, \mathbf{t}, \mathbf{t_{sub}}, \mathbf{s}, \mathbf{t_{rec}}, \mathbf{z}, loc$

Algorithm 2 DenoiseSpikes

Require: Trace $\mathbf{t} \in \mathbb{R}^T$, waveform half size τ, stringency parameter for *adaptive threshold* p_1 and p_2, threshold parameter for *simple threshold* l_1 and l_2, and remaining parameters *params*

$\mathbf{t_s} \leftarrow \textsc{HighPassFilter}(\mathbf{t}, \mathit{params})$ ▷ Remove low frequency baseline

$\mathbf{t_s} \leftarrow \mathbf{t_s} - \textsc{Median}(\mathbf{t_s})$

if $\textsc{UseAdaptiveThreshold} = 1$ **then**

  $\mathbf{s_1} \leftarrow \textsc{AdaptiveThreshold}(\mathbf{t_s}, p_1)$ ▷ see Algorithm 3

else

  $\mathbf{s_1} \leftarrow \textsc{SimpleThreshold}(\mathbf{t_s}, l_1)$ ▷ see Algorithm 4

end if

$\mathbf{q_1} \leftarrow \textsc{Zeros}(T)$ ▷ Create a zero vector with dimension T

$\mathbf{q_1}(\mathbf{s_1}) \leftarrow 1$ ▷ $\mathbf{q}$ is the spike train

$\mathbf{z} \leftarrow \frac{1}{n(\mathbf{s_1})} \sum_{i=1}^{n(\mathbf{s_1})} \mathbf{t_s}(\mathbf{s_1}(i) - \tau : \mathbf{s_1}(i) + \tau)$ ▷ Compute the spike template

$\mathbf{t_s} \leftarrow \textsc{WhitenedMatchedFilter}(\mathbf{t_s}, \mathbf{q_1}, \mathbf{s_1}, \tau)$ ▷ See Algorithm ▷ see Algorithm 5

if $\textsc{UseAdaptiveThreshold} = 1$ **then**

  $\mathbf{s_2} \leftarrow \textsc{AdaptiveThreshold}(\mathbf{t_s}, p_2)$

else

  $\mathbf{s_2} \leftarrow \textsc{SimpleThreshold}(\mathbf{t_s}, l_2)$

end if

$\mathbf{q_2} \leftarrow \textsc{Zeros}(T)$

$\mathbf{q_2}(\mathbf{s_2}) \leftarrow 1$

$\mathbf{t_{rec}} \leftarrow \mathbf{q_2} * \mathbf{z}$ ▷ Convolve the spike train with temporal template to get the reconstructed signal

return $\mathbf{t_s}, \mathbf{s}, \mathbf{t_{rec}}, \mathbf{z}$

Algorithm 3 AdaptiveThreshold

Require: Trace $\mathbf{t_s} \in \mathbb{R}^T$, stringency parameter p, and remaining parameters *params*

$\mathbf{p} \leftarrow \textsc{LocalMaxima}(\mathbf{t_s})$ ▷ Find peak heights of all local maxima
$\mathbf{x} \leftarrow \textsc{LinSpace}(\textsc{Min}(\mathbf{p}), \textsc{Max}(\mathbf{p}), params)$ ▷ Evenly spaced samples between min and max of peak heights
$\mathbf{P_{max}} \leftarrow \text{KDE}(\mathbf{p}, \mathbf{x})$ ▷ Estimated distribution of local maxima at points $\mathbf{x}$
$\mu \leftarrow \textsc{Median}(\mathbf{p})$
$j \leftarrow \textsc{Find}(\mathbf{x}(i) < \mu, \mathbf{x}(i+1) > \mu)$
$\mathbf{P_{noise}} \leftarrow \textsc{Zeros}(\textsc{Len}(\mathbf{P_{max}}))$ ▷ Create a zero vector same size as $\mathbf{P_{max}}$
$\mathbf{P_{noise}}(1:j) \leftarrow \mathbf{P_{max}}(1:j)$ ▷ Estimate noise distribution by symmetrization
if $2j \geq \textsc{Len}(\mathbf{P_{max}})$ **then**
  $\mathbf{P_{noise}}(j+1:\text{end}) \leftarrow \mathbf{P_{noise}}(j:2j - \textsc{Len}(\mathbf{P_{max}}) + 1)$
else
  $\mathbf{P_{noise}}(j+1:2j) \leftarrow \mathbf{P_{noise}}(j:1)$
end if
$\mathbf{F_{max}} \leftarrow \textsc{CumSum}(\mathbf{P_{max}})$ ▷ Cumulative distribution function
$\mathbf{F_{noise}} \leftarrow \textsc{CumSum}(\mathbf{P_{noise}})$
$\mathbf{F_{max}} \leftarrow \mathbf{F_{max}}(\text{end}) - \mathbf{F_{max}}$
$\mathbf{F_{noise}} \leftarrow \mathbf{F_{noise}}(\text{end}) - \mathbf{F_{noise}}$
$\mathbf{g} \leftarrow (\mathbf{F_{max}})^p - (\mathbf{F_{noise}})^p$
$h \leftarrow \mathbf{x}(\textsc{ArgMax}(\mathbf{g}))$
$\mathbf{s} \leftarrow \textsc{LocalMaxima}(\mathbf{t_s}, h)$ ▷ All local maxima with height greater or equal to h
return s

Algorithm 4 SimpleThreshold

Require: Temporal trace $\mathbf{t_s} \in \mathbb{R}^T$, threshold parameter l

$\mathbf{t}' \leftarrow -\mathbf{t_s}(\mathbf{t_s} < 0)$
$\sigma \leftarrow \sqrt{\frac{1}{n(\mathbf{t}')} \sum_{i=1}^{n(\mathbf{t}')} \mathbf{t}'(i)^2}$ ▷ Estimated std based on negative part of the signal $\mathbf{t_s}$
$\mathbf{s} \leftarrow \textsc{LocalMaxima}(\mathbf{t_s}, l \cdot \sigma)$ ▷ Find peaks higher than l times the noise level
return s

Algorithm 5 WhitenedMatchedFilter

Require: Temporal trace $\mathbf{t_s} \in \mathbb{R}^T$, spike train $\mathbf{q} \in \mathbb{R}^T$, spike times $\mathbf{s} \in \mathbb{R}^{n(\mathbf{s})}$, waveform half size τ

$\mathbf{q}' \leftarrow \textsc{Convolve}(\mathbf{q}, \textsc{Ones}(2\tau + 1))$
$\mathbf{t_{noise}} \leftarrow \mathbf{t_s}(\mathbf{q}' < 0.5)$ ▷ The noise signal
$\mathbf{s_n} \leftarrow \textsc{Sqrt}(\textsc{Welch}(\mathbf{t_{noise}}))$ ▷ $\mathbf{s_n}$ is the scaling factor in the frequency domain
$\mathbf{t_s} \leftarrow \text{IFFT}(\text{FFT}(\mathbf{t_s})/\mathbf{s_n})$ ▷ Scale trace in the frequency domain
$\mathbf{z}' \leftarrow \frac{1}{n(\mathbf{s})} \sum_{i=1}^{n(\mathbf{s})} \mathbf{t_s}(\mathbf{s}(i) - \tau : \mathbf{s}(i) + \tau)$ ▷ Compute a spike template based on the prewhitened trace
$\mathbf{t_s} \leftarrow \textsc{CrossCorrelation}(\mathbf{t_s}, \mathbf{z}')$ ▷ Template matching
return $\mathbf{t_s}$

Specific comments:

1. Fig 1: mean image displayed in “back” of subplot b – image is clearly not visible, not clear why it is included. Both images can be plotted on a smaller scale so they will both be visible if the authors want to display both.

Thank you for pointing this out. We updated Fig 1 as suggested:

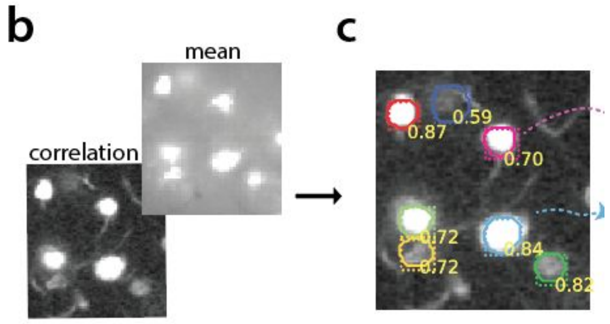

2. Line 63: what do the authors mean by “normalizing by the z-score”? Are they z-scoring each pixel or are they dividing each pixel by the number of standard deviations the value is above/below the mean (this is the definition of the z-score)? Same applies to line 66.

We thank the reviewer for pointing out the confusing definition. We have updated the text to address this point as follows.

Mean image: We averaged the movie across time for each pixel yielding a 2D image. We normalized the 2D image by subtracting the mean of its pixels and dividing by the standard deviation of its pixels. The normalization step enables different datasets to share the same scale as the input to the segmentation step.
Correlation image: The correlation image is a variation of that implemented in [20]. After removing the baseline of the movie by high-pass filtering, we averaged the temporal correlation of each pixel with its eight neighbor pixels yielding another 2D image. The resulting image was then mean-subtracted and divided by its standard deviation.

3. A short description of the motion correction algorithm and its suitability for voltage imaging would make the paper more self-contained.

Following reviewers’ advice, we complemented the motion correction section and discussed the suitability of the same algorithm for voltage imaging.

The motion correction algorithm in *CaImAn* is an efficient implementation of NoRMCorre [23]. NoRMCorre is an online algorithm that uses normalized cross-correlation of each frame with a denoised template to infer shifts. Such shifts can be computed either on the overall frame (rigid motion correction) or on patches of the movie (piece-wise rigid motion correction). The latter case is required when movement is non rigid and a simple translation is not sufficient to compensate for the movement. Such algorithm can in many cases be directly applied to the voltage imaging datasets we have considered, because the frames and templates (see for instance Figure 1e) generally contain high-frequency features. Such features are crucial to precisely identify shifts. Importantly, when this fails we allow the option to apply a high-pass spatial filter to help sharpen such features [16]. In *VolPy* the $gSig\_filt$ parameter controls the size of the kernel for high-pass spatial filtering. We usually inspect visually the results of motion correction, and in all considered cases rigid motion correction was sufficient to capture motion. This might be due to the small size of the field of view (see [23] for a discussion about size of the FOV and its impact on motion correction).

4. Lines 124-125: do the authors mean that instead of a 3-channel RGB image, they are inputting an image that has the mean duplicated to two channels and the correlation in the third? Doesn't this create artifacts as the original network learns features dependent on relations between the color channels that now don't exist?

Thank you for pointing this out. It is correct that the input image has the mean duplicated to two channels and the correlation in the third. Even though the correlations among channels are captured in the original network weights, our results seem to indicate that this problem is attenuated by retraining the last 22 layers of the ResNet (50 layers in total). Mask R-CNN pretrained weights for segmentation tasks are only available for the COCO dataset. Retraining from scratch is not possible in our case considering that we have a reduced training set. We have included a discussion of this point in the text:

translation. Each mini-batch contained six patches. We trained on one GPU the head (the whole network except the ResNet) of the network for 20 epochs (2000 iterations) with learning rate 0.01 and then trained the head together with the last 28 layers of the ResNet for another 20 epochs with learning rate 0.001. We used stochastic gradient descent as our optimizer with a constant learning momentum 0.9. The weight decay was 0.0001. It is possible that correlations among RGB channels existing in the original COCO datasets are not present in our datasets, however retraining some of the ResNet layers is likely compensating for this potential issue.

5. Lines 139-143: this is not clear. Is this magic wand interface to be used to annotate videos in order to then retrain the deep network? Or is it intended to add/remove/correct cells?

We apologize for the lack of clarity. The VolPy GUI we developed, with Python Cell Magic Wand Tool, is used to add/remove/correct cells from the Mask R-CNN outputs (see S2 Vid). Besides, users can choose to bypass the neural networks step and directly input their own masks

annotated through the VolPy GUI or other softwares. We have clarified that in the text as follows, and with added supplements.

While *VolPy* segmentation method achieved good performance on similarly collected datasets, we do not expect it to generalize to completely new datasets out of the box. To overcome this issue, we developed a manual annotation graphical user interface (GUI) tool within *VolPy* to refine the segmentation results. The GUI loads summary images and segmentation results. It enables users to add/delete neurons and save the results for subsequent steps in the *VolPy* pipeline. We equipped the GUI with one-click semi-automatic neuron segmentation based on the Python Cell Magic Wand Tool [27]. See Video V1 and Figure S2 for more details. Segmenting neurons on datasets significantly different from the ones we employed to train Mask R-CNN might lead to poor performance. In this case, users may retrain Mask R-CNN based on a step-by-step guide we provide (`https://github.com/flatironinstitute/CaImAn/wiki/Training-Mask-R-CNN`). Moreover, we also allow users to bypass the Mask R-CNN step and provide their own manual annotations or annotate the data through *VolPy* GUI. This is especially helpful when only few neurons appear in the FOV. We summarize the whole process of segmentation through a flow chart in Figure S3.

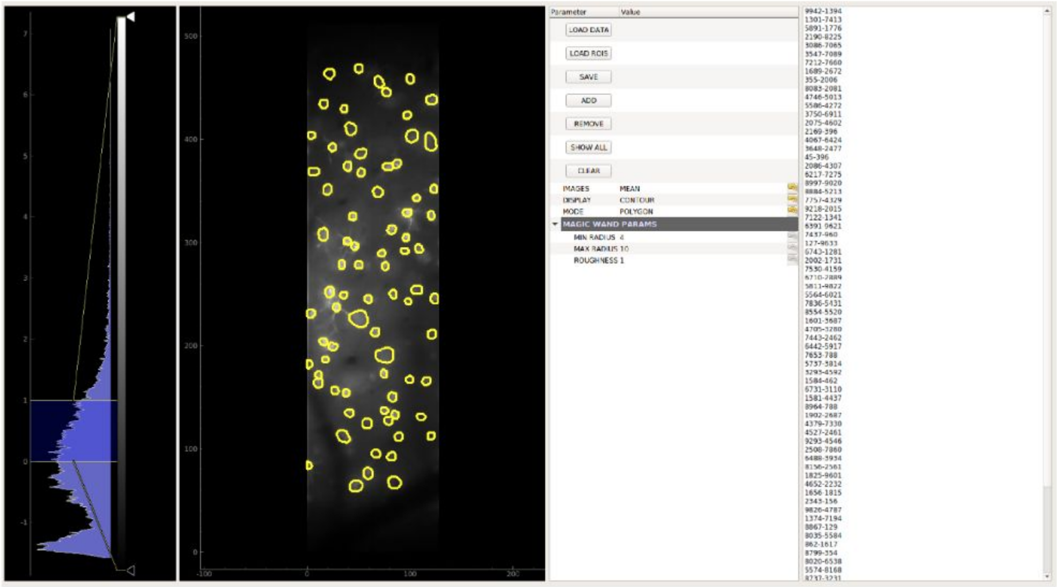

Figure S2 Manual annotation *VolPy* GUI. The interface helps user to select neurons either using polygons (point by point) or a Python implementation of the ImageJ Cell Magic Wand [27]. Users can then remove or add masks, and finally save in hdf5 format the output.

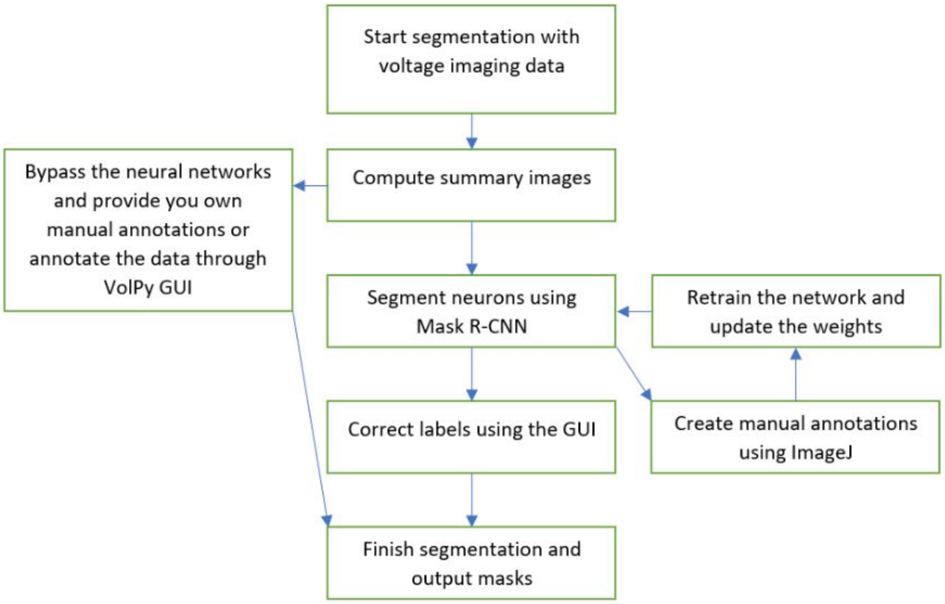

Figure S3 Flow chart for segmentation. Summary images are computed from input voltage imaging movies. Subsequently masks of neurons can be provided in two ways. 1. Neurons can be segmented via a Mask R-CNN neural network trained on the three types of datasets presented in this paper (L1, TEG and HPC). The output labels can be further corrected by the *VolPy* GUI (See Video V1). If users are not satisfied with results of Mask R-CNN, they can manually annotate voltage imaging datasets using ImageJ. Such new annotations can then be used to retrain Mask R-CNN. Details for retraining Mask R-CNN are explained at the page `https://github.com/flatironinstitute/CaImAn/wiki/Training-Mask-R-CNN` 2.Users can also bypasss the Mask R-CNN step and choose to provide their own manual masks labelled either through other softwares or *VolPy* GUI.

6. Line 164: What happens if the dilated background region includes pixels from overlapping neurons?

This is indeed an important problem. We quantified in detail, via simulations, how performance in detecting spikes changed in function of the overlap with other neurons. The results in Fig 4e showed that VolPy is robust to overlaps smaller than about 20%, but then the performance started degrading as the overlap increases, especially for low spike amplitude scenarios.

On separate simulations, we evaluated the performance of *VolPy* in the case of overlapping neurons. We simulated movies with two overlapping neurons and assessed the F_1 score for *VolPy* when varying the degree of overlap (Figure 4e). In terms of spatial footprint extraction, *VolPy* started failing to segment neurons in the large overlapping case (35%). The result could potentially be improved if Mask R-CNN was trained on the simulated datasets as well, especially on neurons featuring similar overlap. Given this, we provided manual masks for testing *VolPy* in such simulated overlapping scenarios. In terms of spike detection, *VolPy* maintained good results when the total overlap was less than 20%. However, the F_1 score dropped as the overlapping area further increased.

e

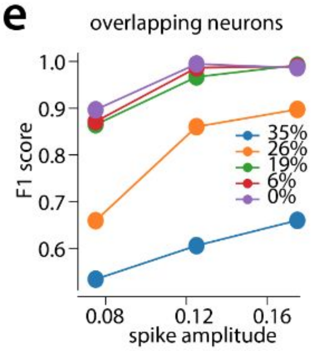

spike amplitude. (e) Evaluation of *VolPy* on overlapping neurons. Average F_1 score detecting spikes in function of spike amplitude and overlap between two neurons.

7. Line 170: can the authors elaborate about a spatial filter w? is this the filter in equation (8)?

Indeed it is. We clarified in the text as follows:

The initial temporal trace $\mathbf{t} \in \mathbb{R}^T$ of the neuron is computed either as the mean of Y_h over the pixels in the ROI, or -- if a spatial filter $\mathbf{w} \in \mathbb{R}^N$ previously calculated is available -- as the weighted average across all pixels in the context region:

$$\mathbf{t} = \begin{cases} \frac{1}{n(S)} \sum_{x \in S} Y_h(x) & \text{if } \mathbf{w} \text{ is not given} \\ Y_h \mathbf{w} & \text{if } \mathbf{w} \text{ is given,} \end{cases} \quad (1)$$

where S denotes set of pixels in the ROI, $n(S)$ denotes the number of pixels in the ROI, $Y_h(x) \in \mathbb{R}^T$ denotes the signal of Y_h at the pixel x. A spatial filter is a matrix of pixel weights which maximizes the amplitude of extracted spikes calculated as a time-varying weighted sum of pixels in the context region. A good spatial filter may be available from processing another chunk of the movie. Compared to simply averaging pixels across the ROI, the initial trace computed with a spatial filter is expected to have better SNR hence enhance the performance of spike detection.

8. Line 201: why is the template time-flipped? Are the authors using correlation?

Exactly, as we further specified in the text, we performed template matching by cross-correlating the spike waveform and the signal. We changed convolution to correlation in the new text to avoid confusion. See answer to Main Comments, point 3.

9. Table 2: number of neurons in the HPC datasets is extremely low given data size. Doesn't this impact the training of the network? Line 240 claims table 2 includes a "train/val" column. What column is this?

Table 2 -> It is actually Table 3. Sorry for the confusion.

Indeed the low number of samples has an effect on training. We have investigated in the paper how the training set size affects the performance of the network on the different datasets. We also have shown how the learning curves correspondingly behave. This is shown in Fig 3d and S4, partially reported below. We also add the following discussion into the text.

We evaluated *VolPy* segmentation performance against a corpus of manually annotated datasets obtained from three human labelers. Humans generally agreed well with a consensus ground truth, with more consistent labeling for easy and high SNR datasets. More difficult datasets, such as HPC, produced controversial annotations and less agreement. On the tested datasets, Mask R-CNN quickly reached asymptotic performance even using small training sets ($\sim$30 neurons), and that performance did not seem to dramatically improve with larger training sets. Our tests suggest that the objective difficulty is the main responsible for performance degradation, with training set size modestly affecting the performance in the tested range. We cannot exclude that a very large corpus of annotated datasets might increase the segmentation performance on difficult datasets, such as HPC. As more dataset become available we plan to further test this possibility.

There were substantial differences in the performance among the three types of datasets for *VolPy*, with L1 obtaining excellent results (close to human annotators) and TEG and HPC progressively worse results (Fig 3c). We hypothesized that two possible sources of variability could account for these differences: the objective difficulty in segmenting the datasets and the number of neurons for training. With regard to segmenting difficulty, the differences among these three types of datasets could be seen clearly through the performance of manual annotators, in which L1 yielded the highest F_1 score and HPC yielded the lowest one, and with the largest variance. With regard to the number of neurons for training, in average 329 neurons in L1 datasets were used for training compared to only 67 and 44 neurons in TEG and HPC datasets respectively (see Tab 3). To test whether differences in the number of neurons for training mainly account for the variability, we separately trained a network for each type of dataset and varied the training set size (i.e. number of neurons for training, see Figure 3d). Although most likely overfitting was present with less than $\sim$100 neurons (See Figure S4), *VolPy* still achieved 0.88 F_1 score on the validation sets of L1 when trained with only 29 neurons. We observed that increasing training set size moderately helped improve *VolPy*'s performance on all three types of datasets. Our results suggest that the objective difficulty accounts for most of the difference in performance within the tested range. However, considering that very small training set size of TEG and HPC were used for training, it is likely that *VolPy*'s performance on these two types of datasets may further increase when trained with more neurons.

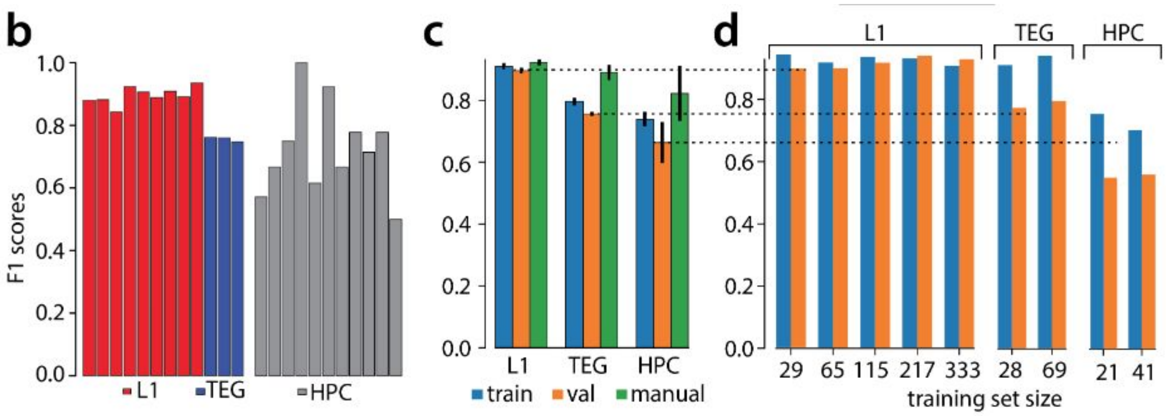

Fig 3. Evaluation of *VolPy* performance. (a) Example of *VolPy* segmentation results against three manually annotated datasets (mouse sensory cortex left, larval zebrafish center, and mouse hippocampus right). Ground truth is built from three different annotations. Matched and mismatched neurons between *VolPy* and ground truth are shown in upper and bottom panels respectively. (b) F_1 scores of *VolPy* for all evaluated datasets. (c) Average F_1 score on training and validation sets grouped by dataset type. Results are provided for training, validation and human annotators (against consensus ground truth) (d) Performance of the network in function of training set size for each dataset type.

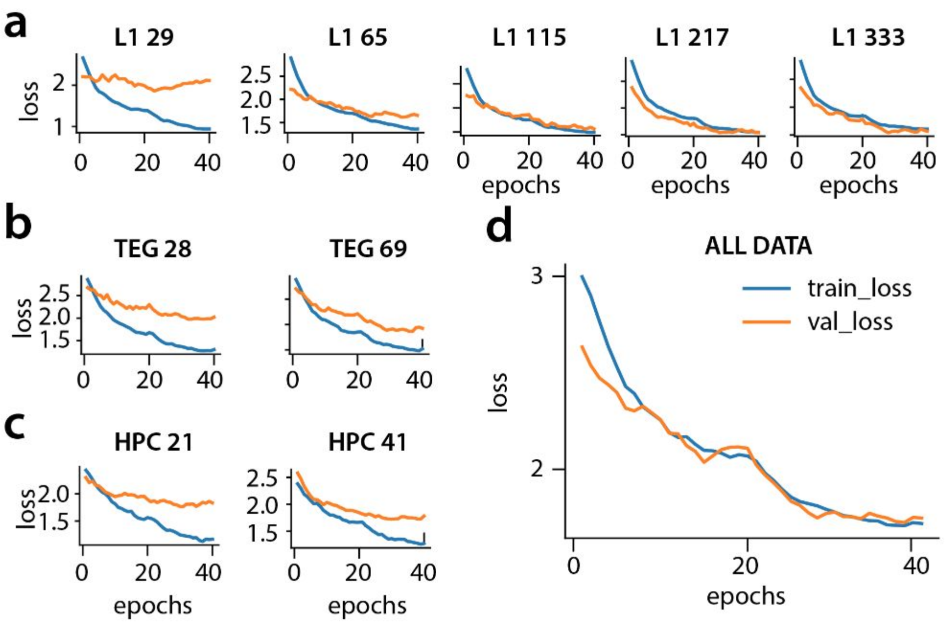

Figure S4 Learning curves in function of dataset size. (a-c) Learning curves corresponding to data in figure 3d. Training (blue) and validation (orange) loss in function of training set size for L1 (a), TEG (b) and HPC (c) datasets. (d) For comparison, learning curves for training and validation set when training on all the datasets.

10. Line 253: can the authors comment on their cost choice for evaluating the spike extraction performance? Why is M=25? The authors should add equations for the Victor-Purpura distance to the paper.

We have adopted a new strategy for spike matching. Given the fact that we only want to compute F1 score/precision/recall, the previous method based on Victor-Purpura distance and Hungarian algorithm is not necessary. Now we use a greedy matching method which is much faster and outputs the same F1 score as the previous method. The details of the algorithm can be found in the paper. In this algorithm we only allow spikes within 10ms to be matched. We chose 10ms based on the fact that neighboring spikes in the electrophysiology ground truth we have evaluated had a minimum interspike interval of 30 ms.

In order to match spikes extracted from simultaneous voltage imaging and electrophysiology datasets, we employed a greedy matching algorithm. Let $\mathbf{v}$ and $\mathbf{e}$ be two sequences of spike times extracted from voltage imaging and electrophysiology datasets respectively. We started by matching the leftmost spike of $\mathbf{v}$ and $\mathbf{e}$. Without loss of generality, we assumed the leftmost spike is $\mathbf{v}(1)$. If the distance between spike $\mathbf{v}(1)$ and its closest spike $\mathbf{e}(1)$ in the other sequence was within 10ms, then two spikes were matched and removed from the sequences; otherwise, spike $\mathbf{v}(1)$ was considered a mismatch and removed from the sequence $\mathbf{v}$. We then started to match the following leftmost spike. This process was repeated until there is no spike in any of these two sequences left. We chose to match spikes within 10 ms based on the fact that neighboring spikes in the electrophysiology had a minimum interspike interval of 30 ms.

After identifying matches and mismatches, we proceeded similarly to what explained above. We defined TP, FP, FN, TN similar to Equation 11:

$$\begin{aligned} &\text{TP} = \text{number of matched spikes} \\ &\text{FP} = \text{number of spikes in } \textit{VolPy} \text{ but not in GT} \\ &\text{FN} = \text{number of spikes in GT but not in } \textit{VolPy} \\ &\text{TN} = 0 \end{aligned} \tag{13}$$

Then we calculated the F_1 score same as Equation 12.

11. Line 275 the authors comment that performance of TEG is “fair” because only 2 datasets were used, and in HPC there are not enough neurons. A more accurate statement should connect the size of the image plane, number of time frames and number of neurons. How much data is necessary to receive good training results?

Thank you for the great point and opportunity for improving the paper. We indeed performed a more accurate analysis of the failure mode for each of the dataset types, as well as an analysis of the network performance in function of the training set size. See also answer to point 9.

12. Figure 3:

Thank you for identifying these issues.

- **subplot a – for HPC is this the best overlay? There are structures in the image that were not annotated but appear bright? Are these not cells?**

  We think that in our previous round of annotations we did miss a few neurons especially in HPC datasets. We think this is because only looking for active neurons in the correlation image and correlation movie was unreliable. Our new annotations selects all neurons in mean and corr image, thereby providing more consistent results. We added more annotators and created a consensus ground truth that seems to capture most of the visible neurons now.

- **What are the vertical red lines that appear within the contours?**

  Vertical lines were the result of a visualization bug that we fixed.

- **For HPC and TEG lines can be made thicker for better visibility. It is hard to see yellow on top of the white cells, perhaps a different color would give better contrast?**
  - We have made the lines for HPC and TEG thicker
  - We have changed the yellow color to green to provide better contrast

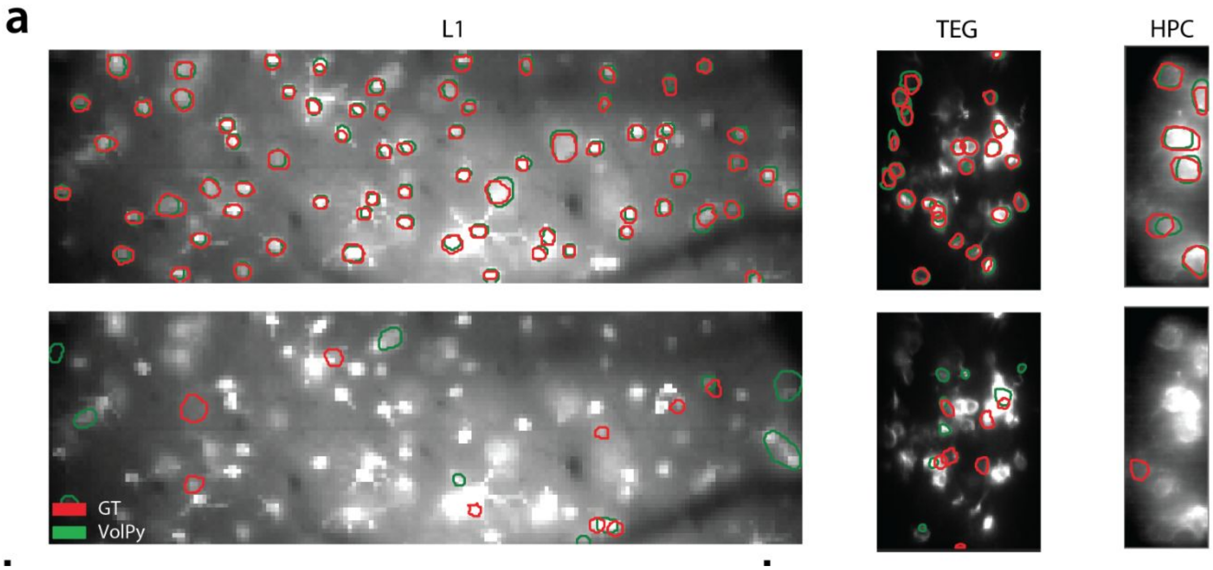

- **Plots (h) and (i) – how do the plots correspond to one another? How many processors were used for plot h? how many time frames are included in data of plot i?**
  - We have updated plots for scalability. All plots for scalability were performed on an L1 movie with 512*128 FOV and 75 selected neurons. The processing time depends on the number of frames and the number of processors used. We controlled one variable and tried to see how processing time changed with the other. in Plot (h) we showed the change of processing time with different number of frames and in Plot (i) we showed the change of processing time with different number of processors used.
  - Plot (h) was performed using 8 processors with 10000, 20000, 40000 frames.
  - Plot (i) was performed on 40000 frames using 1,2,4,8 processors.
  - Fig 3 (h) and (i) are now Fig 6 (a) and (b). The values are now reported in the text at the following location.

Fig 6a reports *VolPy* processing time in function of the number of frames using 8 processors. The results showed that the processing time scales linearly in the number of frames. Processing 75 candidate neurons in the 1.6 minutes long movie (40000 frames) took about 8 minutes. Spike extraction (red bar) was the most time consuming step. In order to probe the benefits of parallelization, we ran *VolPy* 4 times while limiting the available CPUs to 1, 2, 4 and 8 on 40000 frames of the movie (Fig 6b). We observed significant performance gains due to parallelization, especially in the motion correction and spike extraction phase, with a maximum speed-up of 3X.

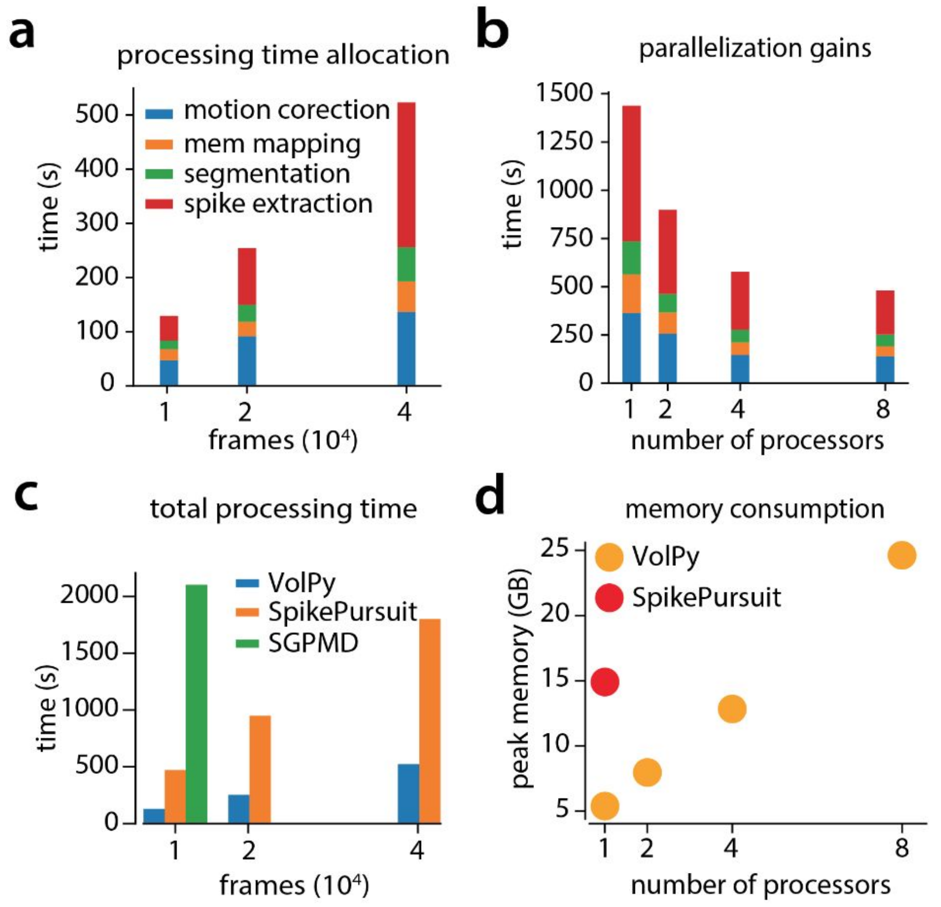

Fig 6. Evaluation of *VolPy* scalability. *VolPy* scalability was evaluated based on a 512x128 pixels movie with 75 annotated neurons. (a) Processing time allocation of *VolPy* with 10000, 20000 and 40000 frames using 8 processors. (b) Processing time of *VolPy* on 40000 frames with 1, 2, 4 and 8 processors. (c) Comparison of performance among *VolPy*(8 processors), SpikePursuit and SGPMD-NMF with 10000, 20000 and 40000 frames. (d) Peak memory usage of *VolPy* and SpikePursuit on 40000 frames. Since *VolPy* supports parallelization we reported memory usage with 1, 2, 4 and 8 processors.

13. Line 321: where were inactive neurons discussed or result presented for these? This is the only place in the text the word "inactive" appears

Thank you, we did move the discussion about inactive neurons to the methods section. We briefly discussed that they fail the locality test and they are automatically removed.

Importantly, inactive neurons are generally identified by the segmentation algorithm, but given the absence of spikes the spatial filters might not match structures internal to the provided masks, thereby failing the locality test. Therefore, inactive neurons with signals not representing the ROI can be removed since they fail locality tests.

14. Missing citations:

Line 113, 123: missing references to deep network architectures,
Line 223: Hungarian algorithm

Thank you for spotting this detail. We added citations for both:

Mask R-CNN (Figure 2a) provides simultaneous object localization and instance segmentation via a combination of two network portions: backbone and head. The backbone features a pre-trained convolutional network (such as VGG [28], ResNet [29], Inception [30]) for feature extraction. Mask R-CNN also exploits another effective backbone: feature pyramid networks [31], a top-down architecture with lateral

In order to measure the performance of *VolPy* segmentation, we compared the spatial footprints extracted by *VolPy* with our manual annotations (see [16] component registration for a detailed explanation). In summary, we computed the Jaccard distance (the inverse of intersection over union) to quantify similarity among ROIs, and then solved a linear assignment problem with the Hungarian algorithm [36] to determine matches and mismatches. Once those were identified, we adopted a precision/recall framework and defined True Positive (TP), False Positive (FP), False Negative (FN), and True Negative (TN) as follows:

15. S3 Video would not play on my computer.

We tested all new formatted videos on Linux, Mac and Windows, and we had success on the three OSs. We used the VLC video player.

Typos:

Thank you for spotting these typos. We addressed all of the mistakes as specified below.

Author summary: "facilitate the process of this..." thisàthese

paper, we present *VolPy*, a software framework that greatly facilitates the preprocessing of this new type of imaging datasets. This pipeline incorporates efficient and optimized

Line 128: “crops” – should patches or sub-images

During training, we randomly cropped the input image into 128x128 patches and applied the following data augmentation techniques using the *imgaug* [33] package: flip, rotation, multiply (adjust brightness), Gaussian noise, shear, scale and translation. Each mini-batch contained six patches. We trained on one GPU the head (the whole

Line 143: is citation [24] the correct citation for magic wand?

The citation for Cell Magic Wand in Python is correct. They first incorporated the Cell Magic Wand into Python. Elsewhere we refer to the appropriate citation for ImageJ Cell Magic Wand.

Guided by these visual cues, three annotators marked the contours of neurons using the FIJI ImageJ Cell Magic Wand tool plugin [21] (Figure S1). Labelers were trained

21. Walker T. Cell magic wand tool. Cell Magic Wand Tool; 2014.

Line 187: Gaussian

Thank you, fixed in several locations

Equation 5: notation is mathematically imprecise. Colon : operator should be defined. This reads like pseudo-code.

Following the reviewer’s advice, we replaced notations that are mathematically imprecise. We removed the Colon operators from our notations.

$$\mathbf{t} = \begin{cases} \frac{1}{n(S)} \sum_{x \in S} Y_h(x) & \text{if } \mathbf{w} \text{ is not given} \\ Y_h \mathbf{w} & \text{if } \mathbf{w} \text{ is given,} \end{cases} \tag{1}$$

where S denotes set of pixels in the ROI, $n(S)$ denotes the number of pixels in the ROI, $Y_h(x) \in \mathbb{R}^T$ denotes the signal of Y_h at the pixel x. A spatial filter is a matrix of pixel weights which maximizes the amplitude of extracted spikes calculated as a time-varying weighted sum of pixels in the context region. A good spatial filter may be available from

$$\mathbf{z}(t') = \frac{1}{n(\mathbf{s})} \sum_{t \in \mathbf{s}} \mathbf{t_s}(t + t') : t' \in [-\tau, \tau], t' \in \mathbb{Z} \tag{7}$$

where $\mathbf{s}$ is the set of spikes, $n(\mathbf{s})$ is the total number of spikes (⑤ in Fig 2b) and τ the waveform half size with default time bin of 20 ms (that is 8 frames if the movie was recorded at 400 Hz). Subsequently, a whitened matched filter [22] is used to enhance spikes with shape similar to the template (⑥ in Fig 2b). This operation is composed of

Line 198: more in details

Thank you, fixed in several locations

Line 283: VolPyspike

Thank you, fixed

==

Reviewer #2:

Voltage imaging with genetically encoded indicators is a powerful emerging technique for measuring neural activity. In this manuscript, Cai et al. describe a suite of computational tools – VolPy – for extracting time series proportional to voltage changes from voltage imaging datasets. VolPy builds upon past algorithmic developments from these authors to provide a scalable pipeline for motion correction, ROI segementation, spike detection, and denoising. This tool efficiently handles large voltage imaging datasets and should be extremely useful for labs establishing voltage imaging as an experimental technique. The authors do a commendable job benchmarking VolPy on several existing voltage imaging datasets and demonstrate generalization to new datasets differing qualitatively from those used for training.

We thank Reviewer 2 for the useful comments, and hope we addressed them fully in this new version of the paper.

Major comments:

1) In the most likely use case, labs implementing VolPy will annotate additional training data and re-train the Mask R-CNN for best results. While the authors include a brief description of this process in the Discussion, the manuscript would benefit from some additional description of the tools included in VolPy for this purpose in the materials and methods and results. I suggest the authors include some quantification of how precision, recall, etc. change with increasing amounts of training data from new datasets differing qualitatively from those used for initial training. This would help give prospective users a better idea of the time investment that would be needed for adoption.

Thank you for pointing out this improvement. In Fig 3d we now evaluate the performance of our algorithm in function of the training set size for different types of datasets. When users have new datasets very different from the datasets we trained, we suggest that users retrain the network. As shown in Fig 3d, to reach a reasonable performance level of Mask R-CNN, users do not need to annotate a large number of datasets.

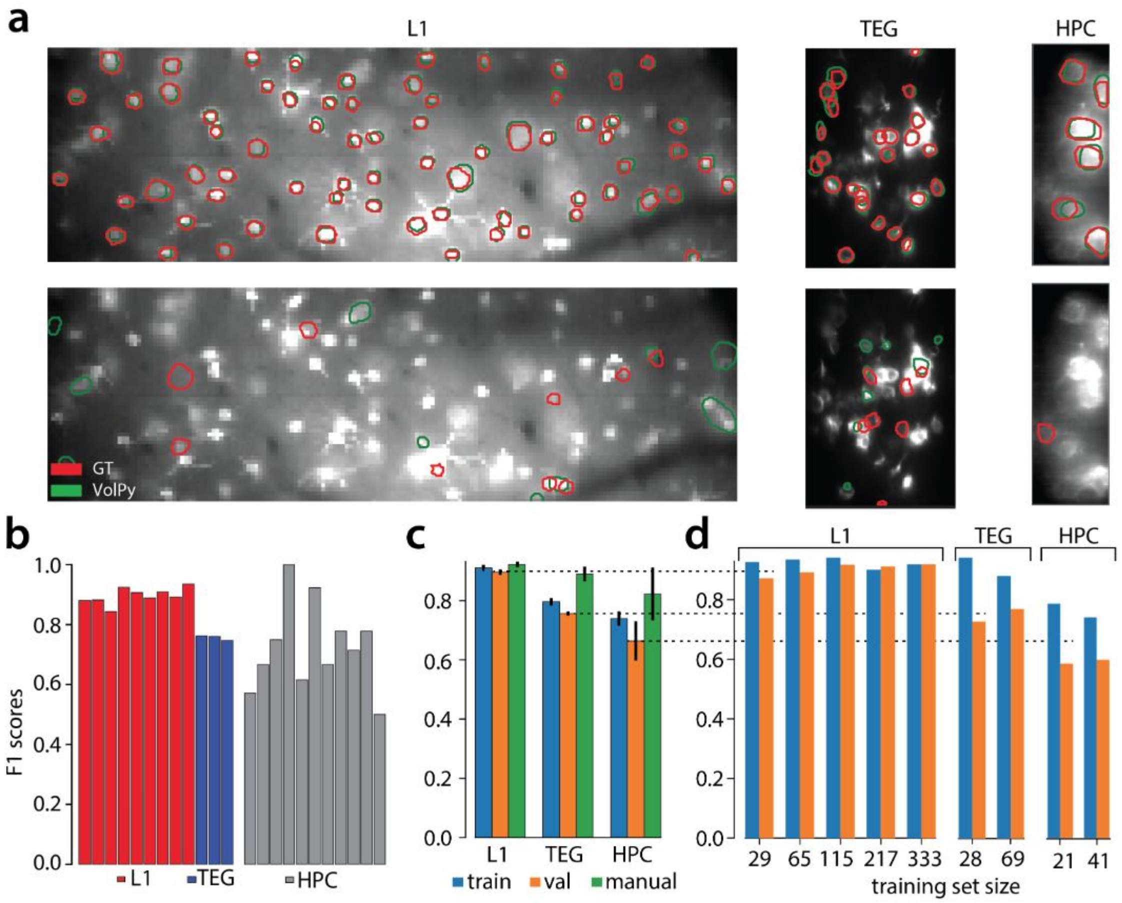

Fig 3. Evaluation of *VolPy* performance. (a) Example of *VolPy* segmentation results against three manually annotated datasets (mouse sensory cortex left, larval zebrafish tegmental area center, and mouse hippocampus right). Ground truth was built from three different annotations. Matched and mismatched neurons between *VolPy* (green) and ground truth (red) were shown in upper and bottom panels respectively. (b) F_1 score of *VolPy* for all evaluated datasets in validation. (c) Average F_1 score on training and validation sets grouped by dataset type. Results were provided for training, validation and human annotators (against consensus ground truth) (d) Performance of the network in function of training set size for each dataset type.

In general we noticed that the performance of Mask R-CNN depends both on the objective difficulty of the dataset (i.e. how easy is to generate consistent annotations), which is quantified in Fig 3c, and on the training set size (Fig 3d). We observe that:

There were substantial differences in the performance among the three types of datasets for *VolPy*, with L1 obtaining excellent results (close to human annotators) and TEG and HPC progressively worse results (Fig 3c). We hypothesized that two possible sources of variability could account for these differences: the objective difficulty in segmenting the datasets and the number of neurons for training. With regard to segmenting difficulty, the differences among these three types of datasets could be seen clearly through the performance of manual annotators, in which L1 yielded the highest F_1 score and HPC yielded the lowest one, and with the largest variance. With regard to the number of neurons for training, in average 329 neurons in L1 datasets were used for training compared to only 67 and 44 neurons in TEG and HPC datasets respectively (see Tab 3). To test whether differences in the number of neurons for training mainly account for the variability, we separately trained a network for each type of dataset and varied the training set size (i.e. number of neurons for training, see Figure 3d). Although most likely overfitting was present with less than ∼100 neurons (See Figure S4), *VolPy* still achieved 0.88 F_1 score on the validation sets of L1 when trained with only 29 neurons. We observed that increasing training set size moderately helped improve *VolPy*'s performance on all three types of datasets. Our results suggest that the objective difficulty accounts for most of the difference in performance within the tested range. However, considering that very small training set size of TEG and HPC were used for training, it is likely that *VolPy*'s performance on these two types of datasets may further increase when trained with more neurons.

In order to facilitate user interaction, we also introduced a new graphical user interface that helps users manually annotate datasets or refine initial estimates from *VolPy* (See S2 Vid). In the methods section we added a detailed explanation of the workflow to train a network from the scratch, or to refine segmentation estimates produced by *VolPy* (See S3 Fig). Below the relevant paper's sections

While *VolPy* segmentation method achieved good performance on similarly collected datasets, we do not expect it to generalize to completely new datasets out of the box. To overcome this issue, we developed a manual annotation graphical user interface (GUI) tool within *VolPy* to refine the segmentation results. The GUI loads summary images and segmentation results. It enables users to add/delete neurons and save the results for subsequent steps in the *VolPy* pipeline. We equipped the GUI with one-click semi-automatic neuron segmentation based on the Python Cell Magic Wand Tool [27]. See Video V1 and Figure S2 for more details. Segmenting neurons on datasets significantly different from the ones we employed to train Mask R-CNN might lead to poor performance. In this case, users may retrain Mask R-CNN based on a step-by-step guide we provide (`https://github.com/flatironinstitute/CaImAn/wiki/Training-Mask-R-CNN`). Moreover, we also allow users to bypass the Mask R-CNN step and provide their own manual annotations or annotate the data through *VolPy* GUI. This is especially helpful when only few neurons appear in the FOV. We summarize the whole process of segmentation through a flow chart in Figure S3.

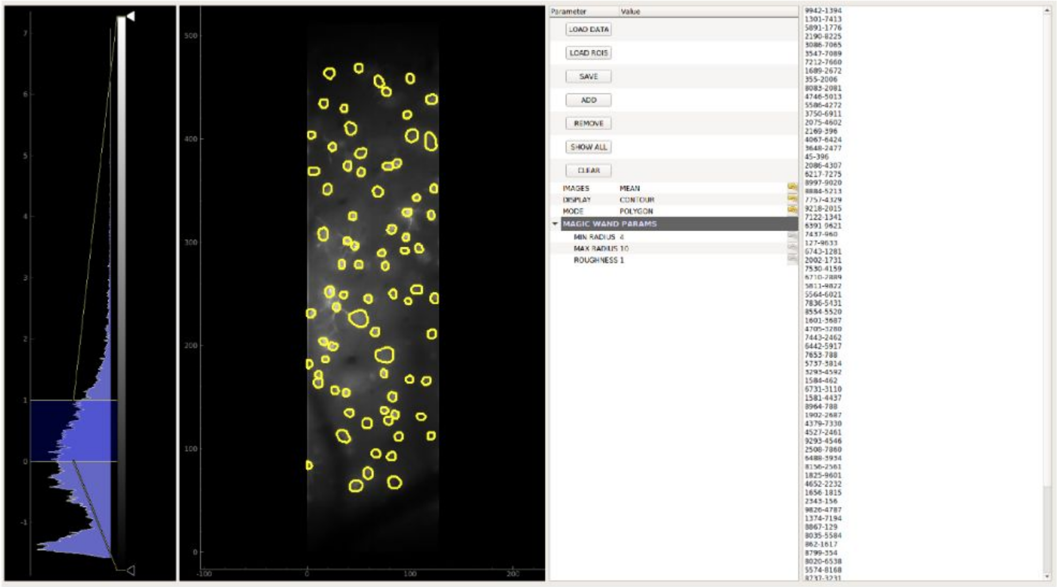

Figure S2 Manual annotation *VolPy* GUI. The interface helps user to select neurons either using polygons (point by point) or a Python implementation of the ImageJ Cell Magic Wand [27]. Users can then remove or add masks, and finally save in hdf5 format the output.

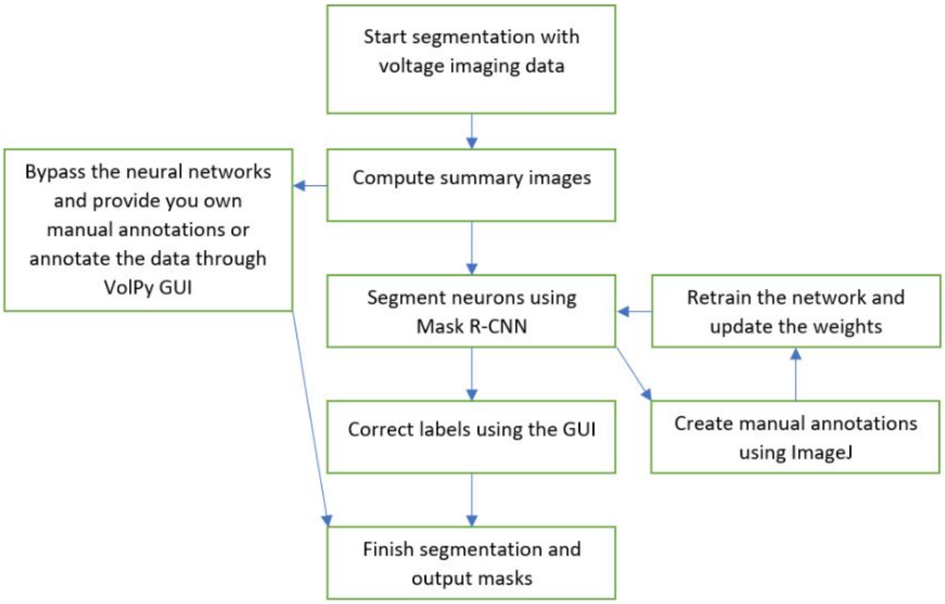

Figure S3 Flow chart for segmentation. Summary images are computed from input voltage imaging movies. Subsequently masks of neurons can be provided in two ways. 1. Neurons can be segmented via a Mask R-CNN neural network trained on the three types of datasets presented in this paper (L1, TEG and HPC). The output labels can be further corrected by the *VolPy* GUI (See Video V1). If users are not satisfied with results of Mask R-CNN, they can manually annotate voltage imaging datasets using ImageJ. Such new annotations can then be used to retrain Mask R-CNN. Details for retraining Mask R-CNN are explained at the page
`https://github.com/flatironinstitute/CaImAn/wiki/Training-Mask-R-CNN`
2.Users can also bypasss the Mask R-CNN step and choose to provide their own manual masks labelled either through other softwares or *VolPy* GUI.

2) The manuscript appears to lack analysis of how spike identification is improved by the modified Spike Pursuit algorithm compared to other simpler approaches.

Thank you for pointing out this weakness. We provided a set of comparisons with other methods. In Figs 4 and 5 we show that VolPy in general outperforms simpler approaches such as taking the average of the region of interest and then thresholding (MeanROI) on simulations (Fig 4) and real data (Fig 5) with and without ground truth. In the same figures, we demonstrate that VolPy outperforms other more complex approaches as well. In the case of SpikePursuit, Fig 4c shows that VolPy mildly outperforms SpikePursuit when both utilize adaptive thresholds, whereas Fig 6c-d demonstrates that the computational performance and scalability of Volpy are about 3-fold improved over SpikePursuit.

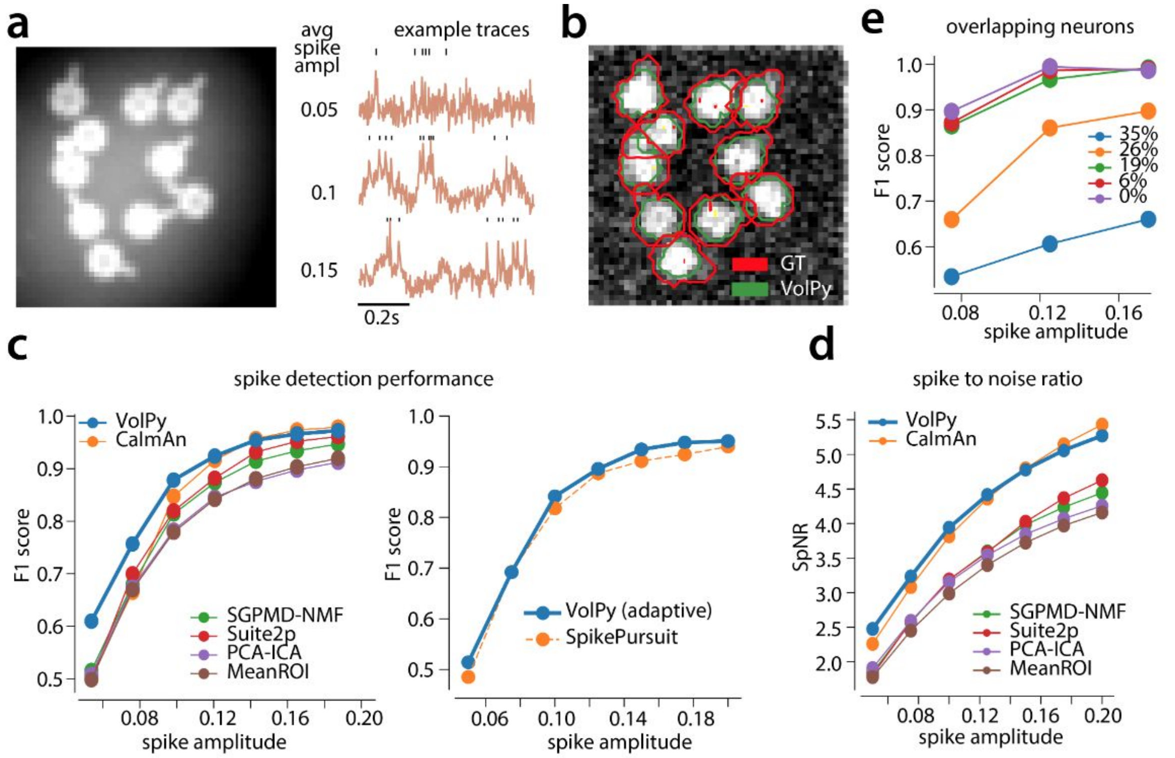

Fig 4. Evaluation of *VolPy* on simulated data. (a) Example of simulated data. Left. Average of movie across time. Right. Three example traces with different average spike amplitude. Higher spike amplitude are associated with higher signal to noise ratio. (b) The result of Mask R-CNN in segmenting the simulated movie (0.1 spike amplitude) laying over the correlation image. (c-d) Performance of *VolPy*, *CaImAn*, SGPMD-NMF, Suite2P, SpikePursuit, PCA-ICA and MeanROI on simulated data. (c) Average F_1 score against ground truth in function of spike amplitude. (Left) All algorithms (including *VolPy*) were evaluated with the optimal threshold. (Right) Comparison with SpikePursuit, *adaptive threshold* in both cases. (d) Spike-to-noise ratio (SpNR) in function of spike amplitude. (e) Evaluation of *VolPy* on overlapping neurons. Average F_1 score detecting spikes in function of spike amplitude and overlap between two neurons.

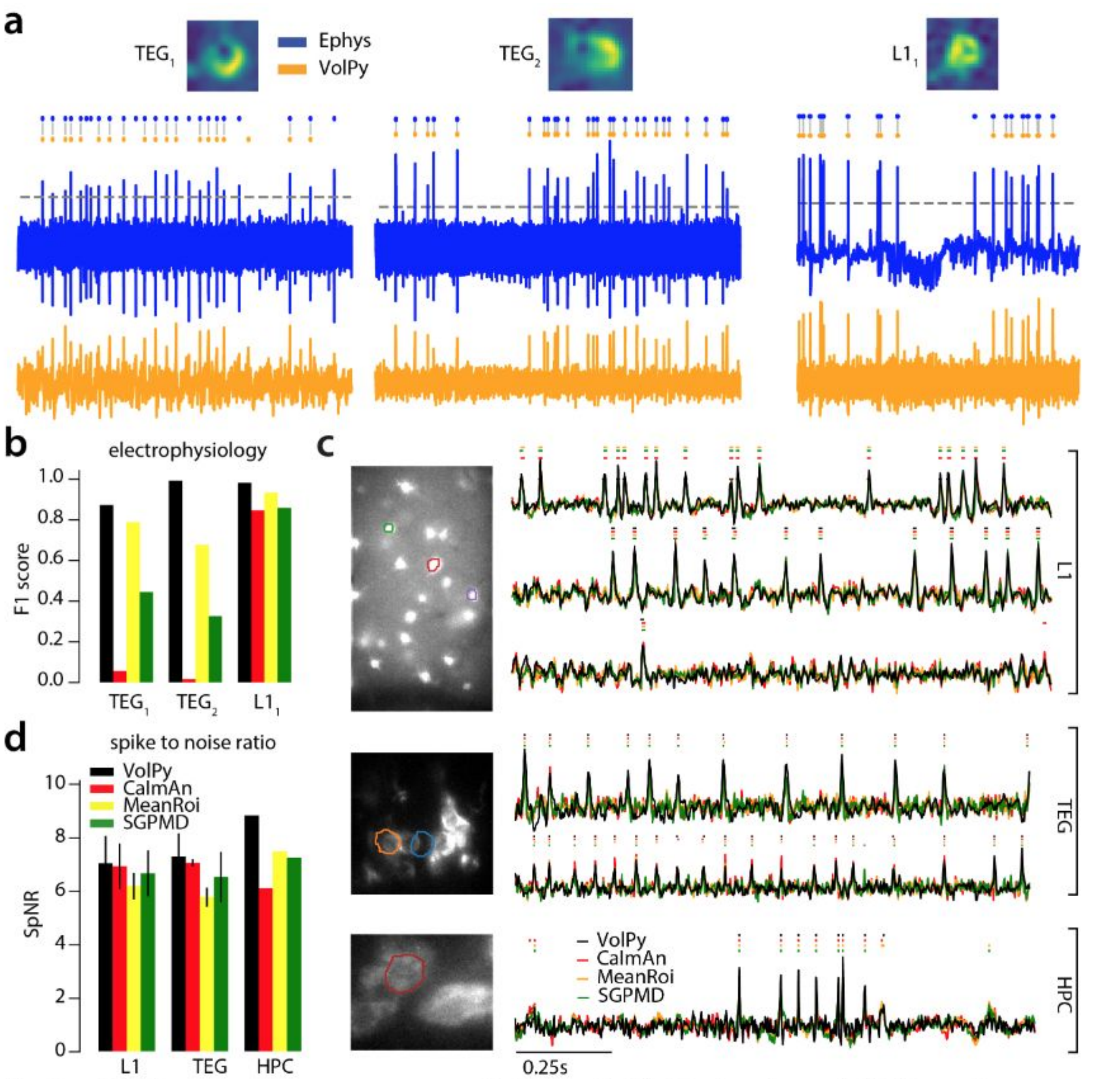

Fig 5. ***VolPy* performance on real data.** (a-b) Evaluation of *VolPy* spike extraction performance against simultaneous electrophysiology. (a) Three neurons, two from larval zebrafish TEG area (TEG_1 and TEG_2) and one from mouse L1 ($L1_1$), for which we had available both electrophysiology and imaging. Top. Spatial footprint extracted by *VolPy*. Middle. Ground truth spikes from electrophysiology (blue) and spikes extracted by *VolPy* (orange), gray vertical lines indicate matched spikes. Bottom. Electrophysiology (blue, top) and fluorescence signal denoised by *VolPy* (bottom, orange). (b) We compared the performance of *VolPy*, *CaImAn*, MeanROI, and SGPMD-NMF in retrieving spikes on the three neurons in (a). (c) Examples of trace extraction results for *VolPy*, *CaImAn*, MeanROI, and SGPMD-NMF. On the left mean image overlaid to example neurons (top L1, middle TEG, bottom HPC). On the right traces and inferred spikes for datasets L1 (top three traces), TEG (traces 4-5 from top) and HPC (bottom trace). (d) Spike to noise ratio (SpNR) for each considered algorithm and dataset type.

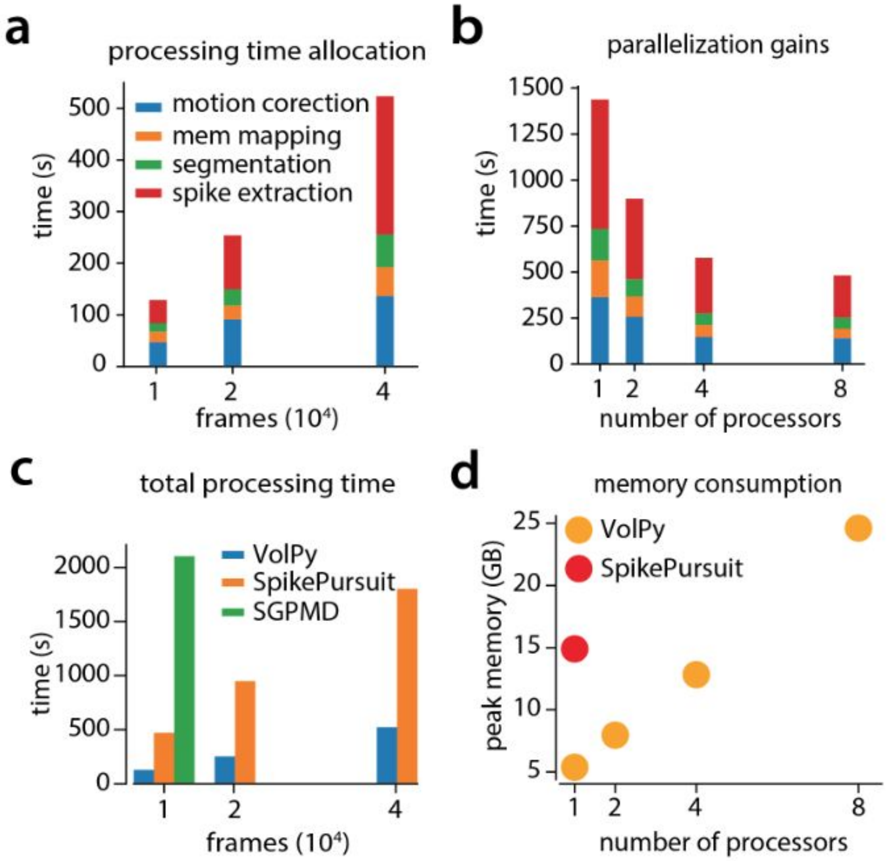

Fig 6. Evaluation of *VolPy* scalability. *VolPy* scalability was evaluated based on a 512x128 pixels movie with 75 annotated neurons. (a) Processing time allocation of *VolPy* with 10000, 20000 and 40000 frames using 8 processors. (b) Processing time of *VolPy* on 40000 frames with 1, 2, 4 and 8 processors. (c) Comparison of performance among *VolPy* (8 processors), SpikePursuit and SGPMD-NMF with 10000, 20000 and 40000 frames. (d) Peak memory usage of *VolPy* and SpikePursuit on 40000 frames. Since *VolPy* supports parallelization we reported memory usage with 1, 2, 4 and 8 processors.

Minor comments:

Line 198: "More in details" appears to be a typo and should be omitted or otherwise corrected.

Thank you, we addressed this imprecision in different points of the paper
