# Supplementary material for: VolPy: Automated and scalable analysis pipelines for voltage imaging datasets
Source: PLoS Comput Biol. 2021 Apr 14;17(4):e1008806. doi: 10.1371/journal.pcbi.1008806 (PMC8075204; doi:10.1371/journal.pcbi.1008806)
Supplement: Attachment — Submitted filename: PLOS COMP BIO VOLPY ROUND 2 REVISION REBUTTAL.pdf [file pcbi.1008806.s012.pdf]

We thank the reviewer and editor for the thorough job and for helping us improve this manuscript.

Reviewer #1: The authors have done an impressive job addressing my comments and questions, improving GT annotations and implementing comparisons to competing methods, highlighting both computational scalability, as well as better performance in extracting spatial footprints and spikes. I recommend the paper be accepted for publication.

Small comment regarding Figure 3 – using red and green contours is probably not an accessible choice for color-blind readers.

We have used the Adobe Illustrator colorblind visualization tool to improve the visibility of all the aspects in Figure 3.

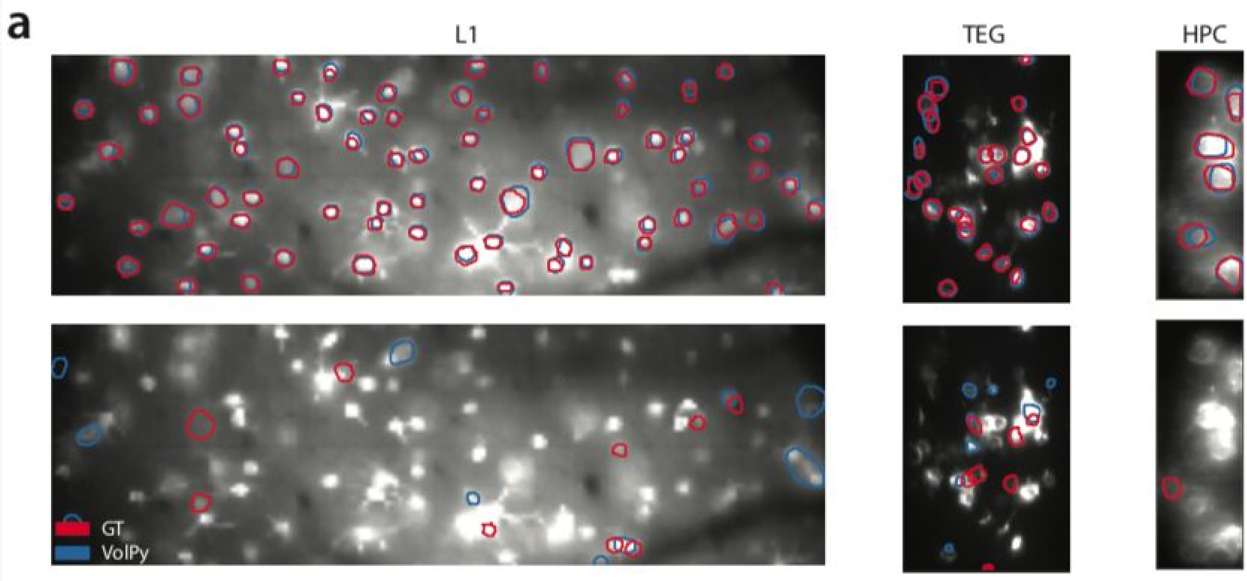


Suggested addition from the editor: Excellent job addressing the concerns of the reviewer and upgrading the paper and the tools. If possible, please add at least a discussion point to make sure readers are aware of fundamental differences between calcium and voltage imaging. Even though in practice it might not be that important, useful signal from voltage sensors are only from the cell membrane, not cytosol like most calcium sensors, and depending on details of the voltage sensor and imaging system (ex. resolution), this may or may not play an important role in how data are extracted/analyzed. I will leave the reviewer's point about figure line colors up to your discretion.

We have addressed this point in the following section:

Common techniques for the analysis of calcium imaging [13] data, a comparable recording modality, have not been systematically tested on voltage imaging datasets and their effectiveness on this new imaging modality is unclear. While signals recorded from calcium imaging are slow and present in the whole cytosol, voltage sensors are mostly expressed on the membrane and produce much faster signals. Therefore, while calcium imaging benefits from averaging in space and time, voltage imaging in general features lower SNR. Further, assumptions made by these methods about underlying signals may [illegible]

Yours sincerely,

Andrea Giovannucci, PhD
